# Modeling and Optimizing Culture Medium Mineral Composition for *in vitro* Propagation of *Actinidia arguta*

**DOI:** 10.3389/fpls.2020.554905

**Published:** 2020-12-23

**Authors:** Radhia Hameg, Tomás A. Arteta, Mariana Landin, Pedro P. Gallego, M. Esther Barreal

**Affiliations:** ^1^Applied Plant & Soil Biology, Department of Plant Biology and Soil Sciences, Faculty of Biology, University of Vigo, Vigo, Spain; ^2^CITACA - Agri-Food Research and Transfer Cluster, University of Vigo, Ourense, Spain; ^3^Departamento de Farmacología, Farmacia y Tecnología Farmacéutica, R+D Pharma Group (GI−1645), Facultad de Farmacia y Agrupación Estratégica en Materiales (AeMat), Universidade de Santiago de Compostela, Santiago, Spain; ^4^Instituto de Investigación Sanitaria de Santiago de Compostela (IDIS), Santiago de Compostela, Spain

**Keywords:** algorithms, artificial intelligence, kiwiberry, modeling, mineral nutrition, plant tissue culture, physiological disorders

## Abstract

The design of plant tissue culture media remains a complicated task due to the interactions of many factors. The use of computer-based tools is still very scarce, although they have demonstrated great advantages when used in large dataset analysis. In this study, design of experiments (DOE) and three machine learning (ML) algorithms, artificial neural networks (ANNs), fuzzy logic, and genetic algorithms (GA), were combined to decipher the key minerals and predict the optimal combination of salts for hardy kiwi (*Actinidia arguta*) *in vitro* micropropagation. A five-factor experimental design of 33 salt treatments was defined using DOE. Later, the effect of the ionic variations generated by these five factors on three morpho-physiological growth responses – shoot number (SN), shoot length (SL), and leaves area (LA) – and on three quality responses - shoots quality (SQ), basal callus (BC), and hyperhydricity (H) – were modeled and analyzed simultaneously. Neurofuzzy logic models demonstrated that just 11 ions (five macronutrients (N, K, P, Mg, and S) and six micronutrients (Cl, Fe, B, Mo, Na, and I)) out of the 18 tested explained the results obtained. The rules “IF – THEN” allow for easy deduction of the concentration range of each ion that causes a positive effect on growth responses and guarantees healthy shoots. Secondly, using a combination of ANNs-GA, a new optimized medium was designed and the desired values for each response parameter were accurately predicted. Finally, the experimental validation of the model showed that the optimized medium significantly promotes SQ and reduces BC and H compared to standard media generally used in plant tissue culture. This study demonstrated the suitability of computer-based tools for improving plant *in vitro* micropropagation: (i) DOE to design more efficient experiments, saving time and cost; (ii) ANNs combined with fuzzy logic to understand the cause-effect of several factors on the response parameters; and (iii) ANNs-GA to predict new mineral media formulation, which improve growth response, avoiding morpho-physiological abnormalities. The lack of predictability on some response parameters can be due to other key media components, such as vitamins, PGRs, or organic compounds, particularly glycine, which could modulate the effect of the ions and needs further research for confirmation.

## Introduction

**FIGURE 1:**
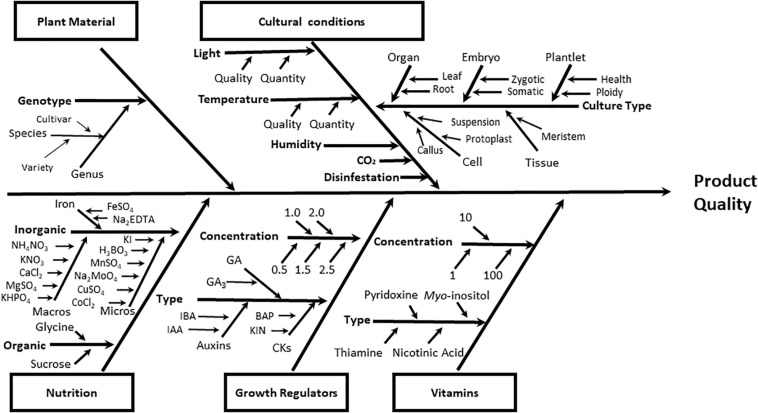
Ishikawa diagram for quick visualization of the main categories of causes (plant material, cultural conditions, nutrition, plant growth regulators, and vitamins) that affect the plant cell tissue culture. Each category grouped factors representing the root causes of variation on the final quality of the process. As example of plant growth regulators. the next auxins (IAA, indole acetic acid; IBA, indole-butiric acid); CKs, cytokinins (BAP, Benzyl adenine purine; KIN, kinetin), and GA, gibberellins (GA_3_, gibberellic acid) were included.

The process of designing protocols for successful plant tissue culture is a very complex task, since there are many potential interacting factors in this process (Figure 1). Plant materials, culture conditions, and culture media ingredients (inorganic and organic nutrients such as carbohydrates, vitamins, and plant growth regulators) are determining factors in the quality of the final product obtained in any plant cell culture protocol (micropropagated seedlings, somatic embryos, doubled haploids, etc.) (Figure 1).

Achieving quality products during *in vitro* plant tissue culture, rather than low survival rates and/or occurrence of physiological disorders, is highly dependent on the mineral nutritional composition of the media, as they are essential for optimal morphogenesis and organogenesis (Ramage and Williams, 2002; George et al., 2008; Sonnewald, 2013). In fact, physiological disorders and/or toxicity due to their deficiency or excess in the culture media inorganic composition has been reported (Bresinsky et al., 2013; Nezami-Alanagh et al., 2019). The inorganic nutrients added into the plant tissue culture media can be differentiated in two groups (Figure 1): macronutrients, taken up in large amounts (>0.5 mM L^–1^) including nitrogen (N), potassium (K), calcium (Ca), phosphorus (P), magnesium (Mg) and sulfur (S); and those used in small quantities or micronutrients (<0.5 mM L^–1^) such as iron (Fe), chlorine (Cl), manganese (Mn), zinc (Zn), boron (B), copper (Cu), and molybdenum (Mo) (Epstein, 1972; George et al., 2008).

The most widely used basal medium, MS (Murashige and Skoog, 1962), although it constitutes a good starting point for the development of new protocols (Niedz and Evens, 2007), is often inadequate because it generates physiological disorders such as shoot tip necrosis and/or hyperhydricity (Nowak et al., 2007; Bhojwani and Dantu, 2013; Nezami-Alanagh et al., 2018). MS mineral composition has been considered as unideal for many fruit species and cultivars (Reed and Hummer, 1995) and even supra-optimal for *in vitro* culture of kiwifruit *Actinidia* sp. (Moncaleán et al., 1999, 2003).

Many strategies have been carried out to improve plant-specific genotypes’ tissue culture protocols by modifying the mineral composition of previously designed media. Initially, a trial and error strategy was employed by the pioneers of plant cell tissue culture (Gautheret, Heller and White’s media) by changing the levels of each factor (independent variable) at a time, named “one factor at time” (OFAT), keeping the rest of the factors constant. Later, Hildebrandt et al. (1946) used the “triangulation method” (three elements varied at time). Finally, Murashige and Skoog (1962) tested the effect of one single element on several concentrations (1, 2, 4, and 8× of the basal medium based on White’s nutrient solution) in the presence of several levels (3×, 8×, 16×, or even 32×) of the remaining elements. However, this strategy presented several disadvantages: (i) it does not give accurate information about the overall optimum, just partial optima for each factor; (ii) it ignores interactions between factors; and (iii) it increases the number of experiments (runs).

Currently, computer-based technologies are able to dramatically reduce the number of experiments and the associated cost (Nezami-Alanagh et al., 2018). As an example, the use of design of experiments (DOE) software facilitates the reduction of the optimal number of treatments to be performed, ensuring adequate sampling of the design space (Niedz and Evens, 2016; Nezami-Alanagh et al., 2019). The analysis of results with computer tools such as the response surface methodology (RSM) have been previously applied to study the composition of plant tissue culture media (Niedz and Evens, 2007; Poothong and Reed, 2016). However, the advantages of artificial intelligence tools, such as neurofuzzy logic, over some statistical analysis, including multiple regression analysis, has been described elsewhere (Landín et al., 2009; Gago et al., 2010a). Advantageously, algorithm-based machine learning (ML) tools provide the ability for autonomous learning and prediction of results without being explicitly programmed or with little human intervention (Gallego et al., 2011). In other words, algorithms can be trained to learn by themselves, generating a model that allows integrating and predicting results. ML approaches, such as artificial neural networks, fuzzy logic, and genetic algorithms, have been proposed as the most up-to-date methodology in the design of culture media (Gago et al., 2010a,c, 2011; Arteta et al., 2018; Nezami-Alanagh et al., 2018) to detect and understand the effect of several factors and their interactions (non-linear and multifactorial) and to predict the optimal combination of salts for the *in vitro* culture of plants (Gago et al., 2011; Gallego et al., 2011).

Due to the level of interest in the kiwi industry to introduce new kiwi genotypes (Kabaluk et al., 1997), our research group has pioneered the establishment of an *in vitro* culture protocol for kiwiberry, particularly for *Actinidia arguta* cv. *Issai* (Hameg et al., 2017). Firstly, based on successful results obtained for kiwifruit tissue culture (Revilla et al., 1992; Paradela et al., 2001), the media Cheng (1975) was used to establish *in vitro* kiwiberry explants (Hameg et al., 2018). Although good performance was achieved, the results suggested that additional research should be done to improve growth responses. To that end, several micropropagation media, previously used for *in vitro* kiwifruit culture, including B5 (Gamborg et al., 1968), Ha (Harada, 1975), Cheng (Cheng, 1975), Kh (Revilla et al., 1992), St (Standardi, 1981), and MS (Murashige and Skoog, 1962), were compared. The most appropriate medium for kiwiberry shoot proliferation was the St medium, but it caused some unwanted physiological disorders, such as basal callus formation (Hameg et al., 2018). For this reason, the challenge of designing a new basal medium that could avoid all these physiological disorders was considered (Hameg, 2019). In this work, we described how the combination of DOE and ML approaches were very useful, as a new strategy, in identifying the multifactorial and non-linear interactions between the culture media mineral nutrients and plant growth responses, and how it is possible to predict its optimal combination for the healthy *in vitro* proliferation of any plant, particularly kiwiberry, using these promising approaches.

## Materials and Methods

### Plant Material and Culture Condition

Nodal segments from a stock culture of *A. arguta* (Sieb. and Zucc.) Planch. ex Miq. cv. ‘Issai’ were maintained in Cheng basal medium (Cheng, 1975) supplemented with 1 mg L^–1^ 6-benzylaminopurine (BAP) and 1 mg L^–1^ gibberellic acid (GA_3_), 8 g L^–1^ agar, and 30 g L^–1^ sucrose. Media pH was set to 5.7 before autoclaving at 121°C for 15 min at 105 KPa (Hameg et al., 2017; Hameg, 2019).

### Experimental Design and Data Acquisition

Salts of MS medium (Murashige and Skoog, 1962) were classified into five independent factors (single salt or group of salts): (i) NH_4_NO_3_, (ii) KNO_3_, (iii) mesos, (iv) micros, and (v) iron. Each factor has several levels corresponding to different concentrations of the MS medium (Table 1). These levels were defined over a range (minimum and maximum) of concentrations expressed as × MS level (1× correspond to MS concentration). The experimental space was designed to decipher the effect of extreme concentrations (very low and high) of ions with levels from 0.1 to 5× MS levels on the morpho-physiological shoots growth and quality responses.

**TABLE 1:** Five factors used to define the five-dimensional design space based on MS medium salts and concentration range expressed as (× MS levels).

Factors	**Media salts**	**Range**
Factor 1	NH_4_NO_3_	0.2–1×
Factor 2	KNO_3_	0.1–1×
Factor 3 (Mesos)	CaCl_2_⋅2H_2_O	0.25–3×
	MgSO_4_⋅7H_2_O	
	KH_2_PO_4_	
Factor 4 (Micros)	MnSO_4_⋅4H_2_O	0.1–1.5×
	ZnSO_4_⋅7H_2_O	
	H_3_BO_3_	
	KI	
	CuSO_4_⋅5H_2_O	
	Na_2_MoO_4_⋅2H_2_O	
	CoCl_2_⋅6H_2_O	
Factor 5 (Iron)	FeSO_4_⋅7H_2_O	1–5×
	Na_2_⋅EDTA	

A five-dimensional experimental design (Niedz and Evens, 2007) was developed using the software Design-Expert^®^ 8 (Design-Expert, 2010). The generated database included 36 treatments. 33 were generated by the software using modified D-optimal criteria (Reed et al., 2013b), while three additional points of MS media were used as controls (34–36; Table 2). All treatments contained MS medium vitamin composition and were supplemented with 2 mg L^–1^ glycine, 30 g L^–1^ sucrose, 8 g L^–1^ agar, ad 1 mg L^–1^ BAP, and 1 mg L^–1^ GA_3_.

**TABLE 2:** Five-factor design with 33 treatments, including three replicates points (6–7, 22–23, and 27–28) plus another three replicates of MS medium (34–36), that are bolded.

**Treatments (media)**	**Factor 1 NH_4_NO_3_**	**Factor 2 KNO_3_**	**Factor 3 Mesos**	**Factor 4 Micros**	**Factor 5 Iron**
1	0.51	0.66	1.65	1.50	3.04
2	1.00	0.66	2.59	0.10	1.00
3	1.00	1.00	2.37	1.50	1.08
4	0.20	0.24	3.00	0.97	1.84
5	0.90	0.10	0.25	0.10	1.60
**6**	**0.20**	**1.00**	**0.94**	**0.10**	**5.00**
**7**	**0.20**	**1.00**	**0.94**	**0.10**	**5.00**
8	0.40	1.00	0.25	1.50	1.00
9	0.81	0.10	0.25	1.40	5.00
10	1.00	1.00	3.00	0.39	5.00
11	0.20	0.33	3.00	0.10	5.00
12	0.81	0.10	0.25	1.40	5.00
13	0.80	0.10	2.99	1.29	1.00
14	0.40	0.10	0.25	0.10	5.00
15	0.20	0.10	2.67	1.50	4.20
16	1.00	0.33	0.25	1.50	1.00
17	0.61	0.64	1.90	0.14	3.54
18	0.20	0.76	0.25	0.10	1.00
19	0.30	1.00	3.00	1.22	5.00
20	1.00	0.10	2.38	0.10	4.46
21	1.00	1.00	0.90	1.50	4.78
**22**	**0.20**	**0.76**	**0.25**	**1.50**	**5.00**
**23**	**0.20**	**0.76**	**0.25**	**1.50**	**5.00**
24	1.00	1.00	0.25	0.10	3.30
25	1.00	0.35	1.52	0.94	3.10
26	0.20	0.76	3.00	1.50	1.00
**27**	**0.24**	**1.00**	**3.00**	**0.10**	**2.00**
**28**	**0.24**	**1.00**	**3.00**	**0.10**	**2.00**
29	0.20	0.76	3.00	1.50	1.00
30	0.32	0.10	2.34	0.10	1.00
31	0.72	0.72	0.90	0.69	1.00
32	0.20	0.10	0.80	1.36	1.00
33	1.00	0.34	3.00	1.50	5.00
**34**	**1.00**	**1.00**	**1.00**	**1.00**	**1.00**
**35**	**1.00**	**1.00**	**1.00**	**1.00**	**1.00**
**36**	**1.00**	**1.00**	**1.00**	**1.00**	**1.00**

Explants about 2 cm were cultured in 200 mL culture vessels containing 30 mL of each medium for 50 days. The cultures were maintained in a growth chamber at 25 ± 1°C under a 16 h photoperiod at 40 μmol m^–2^ s^–1^ irradiation provided by cool white fluorescent tubes (Hameg et al., 2017).

Each treatment included five glass culture vessels (used as replicates) containing three explants each, sealed with plastic caps. The experiments were carried out in triplicate. The explants were harvested after 50 days of culture, all followed, and the next six growth responses were evaluated (Hameg, 2019):

(1) Shoot number (SN), number of new regenerated shoots per explant.
(2) Shoot length (SL), length from the base of the shoot to the tip, per explant (cm).
(3) Leaf area (LA), the sum of areas (cm^2^) of the leaves >1.5 cm. Leaf area per explant was measured using a portable laser leaf area meter (Meter CI-202, CID biosciences, WA, United States).

**FIGURE 2:**
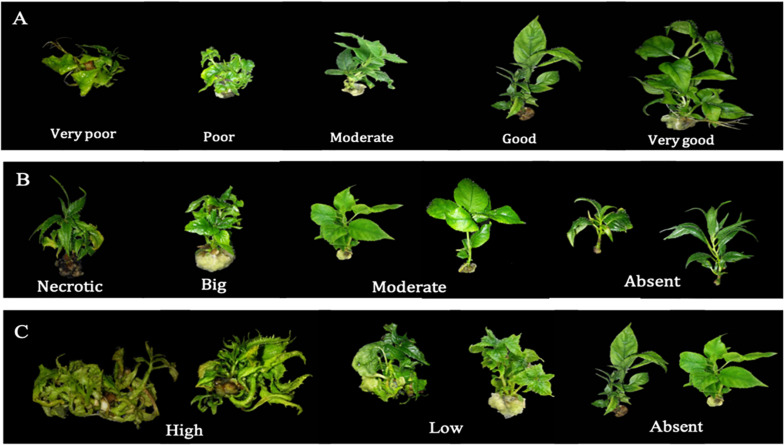
Shoot quality rating **(A)**: 1 (very poor), 2 (poor), 3 (moderate), 4 (good), and 5 (very good); basal callus formation rating **(B)**: 1 (necrotic), 2 (big), 3 (moderate), and 4 (absent) and hyperhydricity rating; **(C)**: 1 (high), 2 (low), and 3 (absent).

As the MS mineral salts have been reported for promoting physiological disorders in some plants, the next three morpho-physiological quality responses were also evaluated:

(1) Shoot quality (SQ), as indicative of shoot vigor, was visually assessed and scored from 1 to 5 (1 very poor, 2 poor, 3 moderate, 4 good and 5 very good; Figure 2A).
(2) Basal callus (BC), callus formation at the cut edge of shoots was visually assessed and scored from 1 to 4 (1 necrotic, 2 big, 3 moderate, 4 absent; Figure 2B).
(3) Hyperhydricity (H), was visually assessed and scored from 1 to 3 (1 high, 2 low, 3 none; Figure 2C).

### Machine Learning Tools for Modeling

Machine learning uses a wide range of algorithms to build mathematical models using databases as training data, helping humans to make predictions and decisions. Here, three artificial intelligence tools were used to build the mathematical models: neural networks (ANNs), fuzzy logic, and genetic algorithms (GA). The commercial neurofuzzy logic software, FormRules^®^ v4.03, (Intelligensys Ltd., United Kingdom) which combines ANNs with fuzzy logic (Colbourn and Rowe, 2005; Landin and Rowe, 2013) was selected to model and decipher the effect of the mineral media composition on plant growth responses, while the commercial software INForm^®^ v5.01 (Intelligensys Ltd., United Kingdom) that combines ANNs with GA (ANNs-GA), was used for the optimization of the mineral nutrition. Advantageously, those artificial intelligence tools allow for the modeling of large databases with an important number of inputs (factors studied) and outputs (plant response parameters determined), independently of the type of data or even if the data set is incomplete, vague, or noisy (Gago et al., 2011; Gallego et al., 2011).

The neurofuzzy logic model was built using 18 inputs (ion concentrations of each treatment) and six outputs (SN, SL, LA, SQ, BC, and H). The ion composition of each treatment (Table 3) was calculated from each salt concentration in the media (Supplementary Table 1). Each ionic concentration was used as an input for the model (Table 3). This procedure deeply facilitates the understanding of the specific effects of mineral elements (ions), avoiding the “ion confounding effect” as described elsewhere (Niedz and Evens, 2006, 2007; Nezami-Alanagh et al., 2017). Instead, the ANNs-GA model was built using 14 inputs corresponding to the MS salts and the same six outputs used for the neurofuzzy logic model (Supplementary Table 1) in order to optimize the salt composition and define a new optimal culture media for kiwiberry.

**TABLE 3:** Mineral nutrients’ (expressed as ions) composition of the different culture media based on the five-factor experimental design (0–33) and response values of the parameters (mean and standard deviation) used to characterize plant growth.

	**Ions (mM)**																		**Growth parameters**			**Quality parameters**		
			
***Media***	**NH_4_^+^**	**NO_3_^–^**	**K^+^**	**Ca^2+^**	**Mg^2+^**	**PO_4_^3–^**	**SO_4_^–2^**	**Cl^–^**	**Fe^2+^**	**BO_3_^–^**	**Mn^2+^**	**Zn^2+^**	**Cu^2+^**	**MoO_2_^2–^**	**Na^+^**	**Co^2+^**	**I^–^**	**EDTA^–^**	**SN**	**SL (cm)**	**LA (cm^2^)**	**SQ**	**BC**	**H**
1	10.47	22.84	14.44	4.95	2.48	2.06	2.98	9.89	0.30	0.15	0.15	0.045	0.00015	0.0015	0.61	0.00016	0.0075	0.30	5.0 ± 0.8	1.6 ± 0.4	20.6 ± 6.3	3.8 ± 0.7	4.0 ± 0.0	2.9 ± 0.5
2	20.61	33.06	15.68	7.74	3.88	3.23	4.00	15.49	0.10	0.01	0.01	0.003	0.00001	0.0001	0.20	0.00001	0.0005	0.10	5.4 ± 2.7	1.7 ± 0.3	40.7 ± 10.9	3.2 ± 1.2	4.0 ± 0.0	1.8 ± 0.9
3	20.61	39.41	21.76	7.09	3.55	2.96	3.86	14.17	0.11	0.15	0.15	0.045	0.00015	0.0015	0.22	0.00016	0.0075	0.11	2.7 ± 0.8	2.3 ± 0.8	26.1 ± 12.8	4.4 ± 0.7	4.0 ± 0.0	2.8 ± 0.5
4	4.21	8.62	8.17	8.98	4.50	3.75	4.81	17.96	0.18	0.10	0.10	0.029	0.00010	0.0010	0.37	0.00010	0.0048	0.18	5.8 ± 2.6	1.9 ± 0.6	12.0 ± 4.0	3.5 ± 0.5	4.0 ± 0.0	1.8 ± 1.0
5	18.55	20.43	2.19	0.75	0.38	0.31	0.55	1.50	0.16	0.01	0.01	0.003	0.00001	0.0001	0.32	0.00001	0.0005	0.16	5.8 ± 1.6	1.2 ± 0.3	7.1 ± 3.4	3.1 ± 0.5	4.0 ± 0.0	3.0 ± 0.0
6	4.12	22.92	19.96	2.81	1.41	1.17	1.92	5.61	0.50	0.01	0.01	0.003	0.00001	0.0001	1.00	0.00001	0.0005	0.50	2.6 ± 1.1	1.2 ± 0.4	6.8 ± 3.2	2.5 ± 0.9	4.0 ± 0.0	3.0 ± 0.0
7	4.12	22.92	19.96	2.81	1.41	1.17	1.92	5.61	0.50	0.01	0.01	0.003	0.00001	0.0001	1.00	0.00001	0.0005	0.50	2.2 ± 1.2	1.4 ± 0.5	7.8 ± 4.4	2.2 ± 0.9	4.0 ± 0.0	3.0 ± 0.0
8	8.25	27.04	19.11	0.75	0.38	0.31	0.67	1.50	0.10	0.15	0.15	0.045	0.00015	0.0015	0.20	0.00016	0.0075	0.10	7.9 ± 2.7	1.1 ± 0.2	8.7 ± 3.9	2.8 ± 0.5	3.5 ± 0.5	2.9 ± 0.3
9	16.73	18.61	2.20	0.75	0.38	0.31	1.06	1.50	0.50	0.14	0.14	0.042	0.00014	0.0014	1.00	0.00015	0.0070	0.50	4.1 ± 1.5	0.7 ± 0.2	3.1 ± 1.7	1.7 ± 0.5	4.0 ± 0.0	3.0 ± 0.0
10	20.61	39.41	22.54	8.98	4.50	3.75	5.05	17.96	0.50	0.04	0.04	0.012	0.00004	0.0004	1.00	0.00004	0.0019	0.50	2.2 ± 1.1	1.3 ± 0.5	9.2 ± 3.6	2.3 ± 1.0	4.0 ± 0.0	2.2 ± 1.0
11	4.21	10.31	9.86	8.98	4.50	3.75	5.02	17.96	0.50	0.01	0.01	0.003	0.00001	0.0001	1.00	0.00001	0.0005	0.50	3.7 ± 1.1	1.0 ± 0.3	6.0 ± 2.1	1.7 ± 0.7	4.0 ± 0.0	1.9 ± 1.0
12	16.73	18.61	2.20	0.75	0.38	0.31	1.06	1.50	0.50	0.14	0.14	0.042	0.00014	0.0014	1.00	0.00015	0.0070	0.50	4.2 ± 1.5	0.8 ± 0.2	3.8 ± 1.2	1.5 ± 0.5	3.1 ± 0.2	3.0 ± 0.0
13	16.57	18.45	5.62	8.94	4.48	3.73	4.75	17.88	0.10	0.13	0.13	0.039	0.00013	0.0013	0.20	0.00014	0.0064	0.10	4.8 ± 1.0	1.7 ± 0.4	17.0 ± 5.5	3.6 ± 0.7	4.0 ± 0.0	1.9 ± 0.7
14	8.16	10.04	2.19	0.75	0.38	0.31	0.89	1.50	0.50	0.01	0.01	0.003	0.00001	0.0001	1.00	0.00001	0.0005	0.50	3.1 ± 1.4	0.7 ± 0.3	3.1 ± 1.3	1.0 ± 0.0	3.0 ± 0.0	3.0 ± 0.0
15	4.12	6.00	5.22	7.99	4.01	3.34	4.62	15.98	0.42	0.15	0.15	0.045	0.00015	0.0015	0.84	0.00016	0.0075	0.42	1.9 ± 0.8	1.1 ± 0.3	4.1 ± 1.8	1.3 ± 0.6	4.0 ± 0.0	2.9 ± 0.3
16	20.61	26.89	6.60	0.75	0.38	0.31	0.67	1.50	0.10	0.15	0.15	0.045	0.00015	0.0015	0.20	0.00016	0.0075	0.10	6.0 ± 1.2	1.5 ± 0.4	14.0 ± 3.4	3.1 ± 0.6	3.9 ± 0.2	2.6 ± 0.7
17	12.66	24.76	14.47	5.69	2.85	2.37	3.23	11.37	0.35	0.01	0.01	0.004	0.00001	0.0001	0.71	0.00001	0.0007	0.35	1.9 ± 1.1	1.2 ± 0.4	8.7 ± 2.3	2.3 ± 0.9	4.0 ± 0.0	3.0 ± 0.0
18	4.12	18.35	14.54	0.75	0.38	0.31	0.49	1.50	0.10	0.01	0.01	0.003	0.00001	0.0001	0.20	0.00001	0.0005	0.10	6.1 ± 1.4	1.3 ± 0.3	8.8 ± 2.8	2.9 ± 0.5	2.0 ± 0.0	2.9 ± 0.3
19	6.26	25.05	22.55	8.98	4.50	3.75	5.16	17.96	0.50	0.12	0.12	0.036	0.00012	0.0013	1.00	0.00013	0.0061	0.50	2.9 ± 1.5	1.7 ± 0.7	14.5 ± 6.4	3.4 ± 1.0	4.0 ± 0.0	2.7 ± 0.8
20	20.61	22.49	4.85	7.13	3.57	2.97	4.03	14.25	0.45	0.01	0.01	0.003	0.00001	0.0001	0.89	0.00001	0.0005	0.45	1.9 ± 0.9	1.5 ± 0.6	6.1 ± 3.7	2.1 ± 0.7	4.0 ± 0.0	2.9 ± 0.3
21	20.61	39.41	19.92	2.68	1.35	1.12	2.02	5.37	0.48	0.15	0.15	0.045	0.00015	0.0015	0.96	0.00016	0.0075	0.48	1.1 ± 0.3	1.7 ± 0.3	5.7 ± 3.4	2.3 ± 1.0	4.0 ± 0.0	3.0 ± 0.0
22	4.12	18.35	14.55	0.75	0.38	0.31	1.07	1.50	0.50	0.15	0.15	0.045	0.00015	0.0015	1.00	0.00016	0.0075	0.50	4.3 ± 0.9	1.0 ± 0.2	4.1 ± 1.4	1.7 ± 0.5	1.0 ± 0.0	3.0 ± 0.0
23	4.12	18.35	14.55	0.75	0.38	0.31	1.07	1.50	0.50	0.15	0.15	0.045	0.00015	0.0015	1.00	0.00016	0.0075	0.50	3.2 ± 1.3	1.0 ± 0.3	4.0 ± 1.6	1.7 ± 0.5	1.2 ± 0.7	3.0 ± 0.0
24	20.61	39.41	19.11	0.75	0.38	0.31	0.72	1.50	0.33	0.01	0.01	0.003	0.00001	0.0001	0.66	0.00001	0.0005	0.33	3.1 ± 1.5	1.3 ± 0.4	4.7 ± 2.0	1.9 ± 0.8	4.0 ± 0.0	2.6 ± 0.9
25	20.61	27.14	8.43	4.53	2.27	1.89	2.71	9.07	0.31	0.09	0.09	0.028	0.00009	0.0010	0.62	0.00010	0.0047	0.31	3.5 ± 0.9	2.2 ± 0.9	16.2 ± 5.3	3.7 ± 0.7	4.0 ± 0.0	2.9 ± 0.5
26	4.12	18.43	18.07	8.98	4.50	3.75	4.80	17.96	0.10	0.15	0.15	0.045	0.00015	0.0015	0.20	0.00016	0.0075	0.10	2.3 ± 1.0	1.9 ± 0.8	21.7 ± 10.3	4.2 ± 0.8	3.6 ± 0.5	2.8 ± 0.4
27	4.95	23.74	22.54	8.98	4.50	3.75	4.72	17.96	0.20	0.01	0.01	0.003	0.00001	0.0001	0.40	0.00001	0.0005	0.20	4.1 ± 1.8	2.0 ± 0.6	28.5 ± 8.5	3.9 ± 0.9	4.0 ± 0.0	1.4 ± 0.7
28	4.95	23.74	22.54	8.98	4.50	3.75	4.72	17.96	0.20	0.01	0.01	0.003	0.00001	0.0001	0.40	0.00001	0.0005	0.20	4.3 ± 1.5	1.9 ± 0.6	29.9 ± 13.8	4.1 ± 0.8	4.0 ± 0.0	1.8 ± 0.7
29	4.12	18.43	18.07	8.98	4.50	3.75	4.80	17.96	0.10	0.15	0.15	0.045	0.00015	0.0015	0.20	0.00016	0.0075	0.10	2.7 ± 1.0	1.8 ± 1.1	18.9 ± 9.3	3.5 ± 1.2	3.7 ± 0.5	2.4 ± 0.8
30	6.51	8.39	4.80	7.00	3.51	2.92	3.63	14.01	0.10	0.01	0.01	0.003	0.00001	0.0001	0.20	0.00001	0.0005	0.10	6.6 ± 2.4	1.1 ± 0.3	9.3 ± 3.9	1.4 ± 0.5	4.0 ± 0.0	1.0 ± 0.0
31	14.92	28.46	14.67	2.68	1.35	1.12	1.54	5.36	0.10	0.07	0.07	0.021	0.00007	0.0007	0.20	0.00007	0.0034	0.10	3.9 ± 1.4	2.6 ± 0.9	28.3 ± 9.3	4.7 ± 0.5	4.0 ± 0.0	2.7 ± 0.5
32	4.12	6.00	2.89	2.39	1.20	1.00	1.48	4.79	0.10	0.14	0.14	0.041	0.00014	0.0014	0.20	0.00014	0.0068	0.10	4.1 ± 1.7	1.2 ± 0.3	5.7 ± 2.9	2.4 ± 0.8	4.0 ± 0.0	2.9 ± 0.3
33	20.61	26.98	10.12	8.98	4.50	3.75	5.20	17.96	0.50	0.15	0.15	0.045	0.00015	0.0015	1.00	0.00016	0.0075	0.50	2.6 ± 2.1	1.5 ± 0.5	10.4 ± 5.5	2.6 ± 0.8	4.0 ± 0.0	2.4 ± 0.9
**MS**	**20.61**	**39.41**	**20.05**	**2.99**	**1.50**	**1.25**	**1.73**	**5.99**	**0.10**	**0.10**	**0.10**	**0.030**	**0.00010**	**0.0010**	**0.20**	**0.00011**	**0.0050**	**0.10**	**3.9 ± 1.3**	**1.7 ± 0.4**	**28.7 ± 9.3**	**4.1 ± 0.4**	**4.0 ± 0.0**	**2.6 ± 0.6**

*Original medium composition (bold) used as control. SN, shoot number; SL, shoot length; LA, leaf area; SQ, shoot quality; BC, basal callus; and H, hyperhydricity.*

Machine learning algorithms were able to build empirical models using the training parameters presented in Table 4. The Adaptive Spline Modeling of Data (ASMOD algorithm) was used by FormRules for the parameter minimization, including in the model the relevant inputs, facilitating a more parsimonious and transparent model for users. Compared to other models of a general structure, ASMOD reduces the model complexity but improves its accuracy even with fewer parameters (Kavli and Weyer, 1994). Finally, ASMOD allows for dividing of the model obtained into submodels to easily interpret the results by generating a set of rules. Once the accuracy of the model was ensured, structural risk minimization (SRM) was selected to obtain models with the highest predictability along with the simplest rules. FormRules^®^ presents the results obtained as a set of linguistic labels or IF-THEN rules with a degree of membership, which greatly facilitates their interpretation. The antecedent part (IF) expresses the conditions at the inputs, and the consequent part (THEN) describes the values of the outputs. The degree of membership represents a degree of truth, ranging from 0 to 1, with 1 meaning that the expected output value is always a complete member of the fuzzy set “low,” “medium,” or “high” (Shao et al., 2006; Gago et al., 2011; Gallego et al., 2011; Nezami-Alanagh et al., 2018).

**TABLE 4:** Train parameter settings for neurofuzzy logic (FormRules^®^ v4.03) and artificial neural networks (INForm^®^ v5.01) software.

**FormRules^®^ v4.03**	**INForm^®^ v5.01**
Minimization parameters (ASMOD)	Number of inputs: 14
Ridge Regression Factor: 1e^–6^	Number of hidden layers: 1
Model Selection Criteria	Number of nodes: 2–4
Structural Risk Minimization (SRM) C1 = 0.868 (except SN: 0.8); C2 = 4.8	Transfer function in hidden layer: asymmetric sigmoid
Number of Set Densities: 2	Output transfer type: linear
Set Densities: 2. 3	Back propagation type: RPROP
Adapt Nodes: TRUE	Targets
Max. Inputs Per SubModel: 4	Target interactions: 1000
Max. Nodes Per Input: 15	Target MS error: 0.0001
	Random seed: 10000
	Test data
	Smart stop: enabled
	Minimum interactions: 20
	Test error weighting: 0.1
	Auto weight
	Interaction overshoot: 200

The Back-Propagation (BP) for Multi-Layer Perceptron (MLP) was used as the training algorithm for InForm^®^ software. To avoid overfitting during MLP training, the data set was split into two groups of data randomly: 80% for training and 20% for testing. Both training error and testing error were checked at every step to prevent overfitting, as described previously (Nezami-Alanagh et al., 2017).

For the optimization process, the software requires the definition of the desirability function for each output, together with their relative importance or weight, expressed on a scale of 0 to 10, 10 being the most important. For media optimization, only the measurable three growth parameters and shoot quality were included. The priority established was: SQ = 10, SN = 9, SL = 8, and LA = 7. As desired values to be achieved, SQ > 4.00, SN > 4.4, SL > 1.6 cm, LA > 28 cm^2^ were included. Finally, the model reveals the percentage of agreement between the predicted values with those desired by the researchers on a scale of 0–100%.

The predictability and accuracy for each parameter developed by both software was assessed using the Train Set R^2^ and the ANOVA f-ratios. Train Set R^2^ values are calculated by the following equation (Shao et al., 2006).

$$R^{2}=\left(1-\frac{\sum_{i=1}^{n}{\left(y_{\text{i}}-y_{\text{i}}^{\prime }\right)}^{2}}{\sum_{i=1}^{n}{\left(y_{\text{i}}-y_{\text{i}}^{"}\right)}^{2}}\right)\times 100\%$$

Where *y*_*i*_ is the experimental point in the data set, *y*_*i*_′ is the predicted point calculated by the model, and *y*_*i*_′′ is the mean of the dependent variable. The higher the Train Set *R*^2^ value, the better the predictability of the model. In previous works, *R*^2^ values higher than 70% have shown good model prediction capacity. It is necessary to avoid *R*^2^ that are too high (>99%), which is indicative of over fitted models of low prediction capacity and should be readjusted as described elsewhere (Colbourn and Rowe, 2005; Landín et al., 2009; Nezami-Alanagh et al., 2017).

To assess statistically significant differences between experimental and predicted values from the model an analysis of variance (one-way ANOVA) was carried out. If the ANOVA *f*-ratio is higher than *f*-critical values for the degrees of freedom of the model, then there are no statistically significant differences between those groups (predicted and experimental values) and the model is accurate.

### Experimental Validation of ANNs-GA Model

In order to validate the model, a new experiment including optimal predicted *R* medium was carried out in the laboratory. As controls, another six media generally used in kiwifruit tissue culture, such as MS, B5, St, and Ha, were also tested. A total of 45 explants per medium were cultivated in the same conditions as above. After 50 days, the growth parameters (SN, SL, and LA) and quality parameters (SQ, BC, and H) were recorded.

Statistical analysis was used to check the validity of the new optimized *R* medium and search for the significant differences between the new *R* medium and MS. Continuous data (SL and LA) were analyzed using ANOVA with Tukey’s Studentized range, (HSD) *post hoc* test at α = 0.001. Discrete (SQ, BC, and H) and categorical (SN) data were analyzed by the non-parametric Kruskal–Wallis test at α = 0.001. All the statistical analyses were performed using Statistica v.12 (StatSoft-Inc., 2014).

## Results

### Effect of Mineral Nutrition on Shoot Growth and Quality Responses

The computer-based software for optimal design was used to guarantee a well sampled design space, giving a database of 34 treatments combining all 18 mineral nutrients based on the MS medium composition (Table 3). To focus on the effect of the mineral composition, the other media ingredients were kept constant. The MS based treatments caused a great variety of physiological responses compared with the MS control (Table 3). As example, very good quality (#31: 4.7), large SN (#8: 7.9), long SL (#31: 2.6 cm), and very large LA (#2: 40.7 cm^2^) versus the MS (4.1, 3.9; 1.7 cm and 28.7 cm^2^, respectively) were obtained. On the contrary, very poor quality (#14: 1.0), few SN (#21: 1.1), short SL (#9: 0.7 cm), or LA (#9 and 14: 3.1 cm^2^) were also achieved.

**FIGURE 3:**
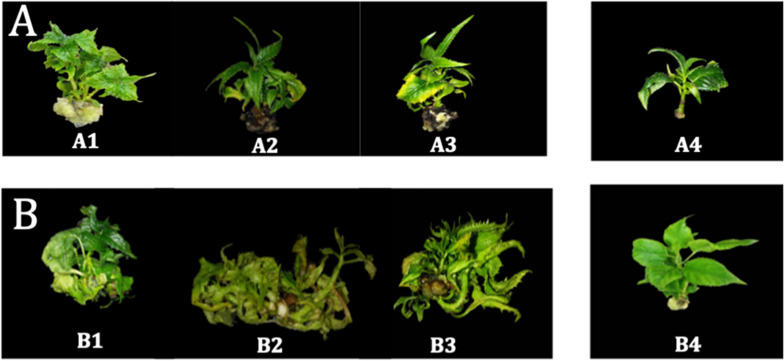
Morpho-physiological abnormalities responses to some media treatments as basal callus **(A)**: treatments 18 **(A1)**, 22 **(A2)**, and 23 **(A3)** and hyperhydricity **(B)**: treatments 2 **(B1)** and 30 **(B2,B3)** versus high quality plants without symptoms **(A4,B4)**.

Physiological disorders such as necrotic, big, or even moderate basal callus and hyperhydricity were also found (Figure 3). As an example, the treatments #18, 22, and 23 (Figures 3A1–A3) promoted the formation of big and necrotic callus at the cut edge of shoots, whereas the treatments #2, 3, and 30 (Figures 3B1–B3) promoted hyperhydricity. The MS medium, used as control, did not show basal callus formation (4.0) on kiwiberry, but some hyperhydricity (1.4) was detected.

Classical statistical techniques (ANOVA) are very useful in data analysis, but they do not help much in extracting valuable information about key mineral nutrients in these complex processes, or the right combination of minerals to promote ever-healthy plantlets. Currently, other advanced algorithm-based technologies like decision trees, SRM, or machine learning tools look promising for deciphering key minerals. Here, the latest technologies were used.

### Neurofuzzy Logic Models

The neurofuzzy logic tool was able to successfully model the dataset (Table 3) with the training parameters described in Table 4. The results of the Train Set *R*^2^, higher than 70% for all parameters, indicated good performance and a high predictability of the neurofuzzy logic models (Table 5). Moreover, the ANOVA f ratio was always higher than the f critical value showing the quality and accuracy for prediction, since no statistically significant differences (α < 0.001) between experimental and predicted values were found (Table 5 and Supplementary Figure 1).

**TABLE 5:** Neurofuzzy logic model Mean Square Error (MSE), train set *R*^2^, ANOVA parameters for training [*f-*ratio, degrees of freedom (df1: model and df2: total), and *f-*critical value for α = 0.05].

**Outputs**	**MSE**	**Train set *R*^2^ (%)**	***f-ratio***	**df1**	**df2**	***f****-***critical (α = 0.05)**	**Critical factors**
SN	0.0055	89.495	9.030	16	33	3.558	**Na^+^ × MoO_2_^2–^** SO_4_^2–^ × I^–^ NH_4_^+^ × NO_3_^–^ × BO_3_^–^
SL	0.0107	80.063	10.350	9	33	4.255	Fe^2+^ K^+^ **Mg^2+^** BO_3_^–^ PO_4_^3–^
LA	0.0159	75.629	17.378	5	33	5.382	**NO_3_**^–^ × **Na^+^** Cl^–^
SQ	0.0050	92.998	26.563	11	33	3.975	Fe^2+^ **K^+^** × **SO_4_^2^**^–^ BO_3_^–^ NO_3_^–^
BC	0.0069	89.346	16.771	11	33	3.975	**PO_4_^3^**^–^ × **NH_4_^+^**
							SO_4_^–2^
H	0.0173	75.250	11.289	7	33	4.675	**Cl**^–^ × **I**^–^**SO_4_^2–^ × BO_3_^–^**

*Critical factors (significant inputs) computed for each output are bolded. SN, shoot number; SL, shoot length; LA, leaf area; SQ, shoot quality; BC, basal callus; and H, hyperhydricity.*

Neurofuzzy logic approach also succeeded in identifying the key factors (inputs) for each parameter (outputs) studied. Among the 18 evaluated ions, just 11 were critical and explain variations among treatments (Table 5). Of these, five belong to macronutrients (N, K, P, Mg, and S) and six to micronutrients (Cl, Fe, B, Mo, Na, and I). While some ions caused independent effects, others interacted with each other. As an example, the variability of the SN parameter was explained as a function of the interactions of seven ions, Na^+^ × MoO_2_^2–^; SO_4_^2–^ × I^–^ and NH_4_^+^ × NO_3_^–^ × BO_3_^–^, while the SL variability was independently affected by the following 5 ions: Fe^2+^, K^+^, Mg^2+^, BO_3_^–^, and PO_4_^3–^ (Table 5).

#### Morpho-Physiological Growth Responses

The variability on the new regenerated shoots per explant (SN parameter) is explained by three submodels showing three interactions of Na^+^ and MoO_2_^2–^ (strongest effect; submodel 1), the SO_4_^2–^ × I^–^ interaction (submodel 2), and NH_4_^+^ × NO_3_^–^ × BO_3_^–^ (submodel 3; Table 5).

Through simple rules IF THEN the model pinpoints the negative effect of both Na^+^ and MoO_2_^2–^ ions at high concentrations on shoot proliferation (Table 6; rule 4), recommending their use at low concentrations to obtain the highest number of regenerated shoots (Table 6; rule 1). In both cases, the membership was 1.00 which means that if we combine low concentrations of both ions, we will obtain a truly high number of new shoots.

**TABLE 6:** Rules for morpho-physiological growth responses (SN, shoot number; SL, shoot length and LA, leaf area) with their membership degree (MD) generated by neurofuzzy logic.

**Rules**		**[NH_4_^+^]**	**[NO_3_^–^]**	**[K^+^]**	**[Mg^2+^]**	**[PO_4_^3–^]**	**[SO_4_^2–^]**	**[Cl^–^]**	**[Fe^2+^]**	**[BO_3_^–^]**	**[MoO_2_^2–^]**	**[Na^+^]**	**[I^–^]**		**SN**	**SL**	**LA**	**MD**
**1**	**IF**										**Low**	**Low**		**THEN**	**High**			**1.00**
2											High	Low			Low			1.00
3											Low	High			High			1.00
**4**											**High**	**High**			**Low**			**1.00**
5							Low						Low		Low			1.00
6							Low						Mid		Low			1.00
7							Low						High		High			1.00
8							High						Low		Low			1.00
9							High						Mid		High			1.00
10							High						High		High			1.00
11		Low	Low							Low					Low			1.00
12		Low	Low							High					High			1.00
13		High	Low							Low					Low			1.00
14		High	Low							High					High			1.00
15		Low	High							Low					Low			1.00
16		Low	High							High					High			1.00
17		High	High							Low					Low			1.00
18		High	High							High					High			1.00
19	**IF**								Low					**THEN**		High		1.00
20									High							Low		0.94
21				Low												Low		0.74
22				High												High		1.00
**23**					**Low**											**Low**		**1.00**
**24**					**Mid**											**High**		**1.00**
25					High											Low		1.00
26										Low						Low		1.00
27										Mid						High		1.00
28										High						Low		0.99
29						Low										High		1.00
30						Mid										Low		1.00
31						High										High		1.00
**32**	**IF**		**Low**									**Low**		**THEN**			**Low**	**1.00**
**33**			**High**									**Low**					**High**	**1.00**
34			Low									High					Low	1.00
35			High									High					Low	1.00
36								Low									Low	0.93
37								High									High	0.62

*The inputs with the strongest effect indicated by the model are bolded. SN, shoot number; SL, shoot length; LA, leaf area; and MD, membership degree.*

Also, the presence of High SO_4_^2–^ concentrations in the medium in combination with mid or high I^–^ concentration significantly increases the number of shoots (Table 6; rules 9–10). Finally, the model also pinpoints the key role of High concentrations of BO_3_^–^ combined with either low or high concentrations of NH_4_^+^ × NO_3_^–^ in the culture medium (Table 6; rules 12, 14, 16, and 18).

Shoots length (SL) of *A. arguta* is independently influenced by the effect of five ions: Fe^2+^, K^+^, Mg^2+^, BO_3_^–^, and PO_4_^3–^ (Table 5). The model selects Mid Mg^2+^ as the adequate concentration to obtain large (high) shoot length (Table 6; rules 24). When low (rule 23) or high (rule 25) concentrations are added to the media (membership 1.00) a truly short SL is achieved. Additionally, Low Fe^2+^, High K^+^, Mid BO_3_^–^, and Low or High PO_4_^3–^ concentrations in the culture media increases SL (Table 6; rules 19, 22, 27, 29, and 31).

Area of leaves in *A. arguta* were mainly influenced by the interaction of NO_3_^–^ and Na^+^ concentrations and the single effect of Cl^–^ concentration (Table 5). The greatest leaf area is achieved with the combination of High NO_3_^–^ and Low Na^+^ concentrations (Table 6; rule 33). Other combinations promoted worse results for leaf area (Table 6, rules 32, 34–36). High values of LA are also obtained when high concentrations of Cl^–^ were used (Table 6, rule 37).

#### Morpho-Physiological Quality Responses

Mineral nutrients had a great impact on the appearance of morpho-physiological abnormalities such as basal callus and hyperhydricity (Table 3 and Figure 3). Therefore, a parameter to establish the quality of the shoots was included as output and determined as explained previously (Figure 2A).

The neurofuzzy logic software selected just five ions as critical factors for the quality of the shoots: the interaction between K^+^ and SO_4_^2–^ as the stronger, and the independent effect of Fe^2+^, BO_3_^–^, and NO_3_ (Table 5). The best shoot quality is promoted (membership 1.00), if High K^+^ and Mid SO_4_^2–^ concentrations are added into the media (Table 7; rule 44), although High SO_4_^2–^ concentration also improves shoot quality with high membership (0.83; Table 7; rule 45). On the contrary, the high concentration of Fe^2+^ ion promotes a truly low quality of shoots (Table 7; rule 39). The same occurs when the concentrations of BO_3_^–^ and NO_3_^–^ are high (Table 7; rules 48 and 51). However, if mid concentration of BO_3_^–^ and NO_3_^–^ ion are used, a truly high shoot quality will be obtained (Table 7; rules 47 and 50) with the highest membership degree (1.00). As an example, these results agree with the treatments #3 and 26 (Table 3).

**TABLE 7:** Rules for morpho-physiological quality responses (SQ, shoot quality; BC, basal callus; H, hyperhydricity) with their membership degree (MD) generated by neurofuzzy logic.

**Rules**		**[NH_4_^+^]**	**[NO_3_^–^]**	**[K^+^]**	**[PO_4_^3–^]**	**[SO_4_^–2^]**	**[Cl^–^]**	**[Fe^2+^]**	**[BO_3_^–^]**	**[I^–^]**		**SQ**	**BC**	**H**	**MD**
38	**IF**							Low			THEN	High			1.00
39								High				Low			1.00
40				Low		Low						Low			0.84
**41**				**Low**		**Mid**						**Low**			**1.00**
42				Low		High						Low			0.62
43				High		Low						Low			1.00
**44**				**High**		**Mid**						**High**			**1.00**
45				High		High						High			0.83
46									Low			Low			1.00
47									Mid			High			1.00
48									High			Low			0.93
49			Low									Low			1.00
50			Mid									High			1.00
51			High									Low			0.58
**52**	**IF**	**Low**			**Low**						**THEN**		**Low**		**1.00**
53		Mid			Low								Low		1.00
54		High			Low								Low		1.00
**55**		**Low**			**Mid**								**High**		**1.00**
56		Mid			Mid								High		0.92
57		High			Mid								High		1.00
58		Low			High								High		0.79
59		Mid			High								High		1.00
60		High			High								High		0.89
61						Low							High		1.00
62						Mid							High		0.75
63						High							High		1.00
64	**IF**						Low			Low	**THEN**			Low	0.60
**65**							**Low**			**High**				**High**	**1.00**
**66**							**High**			**Low**				**Low**	**1.00**
67							High			High				High	1.00
68						Low			Low					High	1.00
69						Low			High					Low	1.00
70						High			Low					High	1.00
71						High			High					Low	1.00

*The inputs with the strongest effect indicated by the model are bolded. SQ, shoot quality; BC, basal callus; H, hyperhydricity; and MD, membership degree.*

The neurofuzzy model was also able to identify the key ions causing the physiological abnormalities described here (Table 5) and explain their effect using simple rules (Table 7). Thus, the model pinpoints the effect of only three ions on the development of basal callus: PO_4_^3–^ × NH_4_^+^ and SO_4_^2–^ (Table 5). The model highlights the positive effect of using Mid to High PO_4_^3–^ concentration (Table 7; Rules 55–60), which favor the production of healthy shoots that show moderate or absent callus formation (Figure 2B), particularly at Low NH_4_^+^ concentration (Table 7, rule 55; membership 1.00). If Low PO_4_^3–^ is added to the media, (membership 1.00) the formation of big and truly necrotic basal callus are promoted (Table 7; rules 52–54), independently of the NH_4_^+^ concentration. Finally, the rules 61–63 generated by the model shows that the presence of SO_4_^2–^ at any concentration within the design space reduces the basal callus formation, generating healthy shoots (Table 7).

The hyperhydricity, a well-described physiological disorder in plant tissue culture, is associated in *A. arguta* to the combined effect of four ions: Cl^–^ × I^–^ and SO_4_^2–^ × BO_3_^–^ (Table 5). High I^–^ concentration in the medium promoted no hyperhydricity (Table 7; rule 65 and 67), with a stronger effect if combined with low concentration of Cl^–^ (rule 65). Interestingly, Low BO_3_^–^ concentration also minimized the hyperhydricity (Table 7; rules 69 and 71), in interaction with any concentration of SO_4_^2–^.

### Designing New Optimized Medium and Experimental Validation

A database including as inputs the salt concentrations in the different culture media and as outputs all growth and quality parameter results (Supplementary Table 1) was modeled using INForm^®^ software, achieving high predictability. Both the Train Set and Test Set *R*^2^ were above 70% (76.66 < *R*^2^ < 96.59) for all the parameters studied (Supplementary Table 2).

Genetic algorithms predicted the best combination of salts that would provide, simultaneously, the highest values for all parameters: 4.8, 2.6 cm, 39.5 cm^2^ and 4.4 for the SN, SL, LA, and SQ, respectively. The new optimized medium, named “R medium,” predicted higher values than MS medium used as control (Table 3).

The results from the experiment carried out in order to validate “R medium” are shown in Table 8. As can be seen, predicted and experimental (Table 8) are close for both *R* and MS medium. In fact, no significant differences (α < 0.001) between the data obtained in both experiments for MS media (Tables 3, 8), used as internal control, was detected.

**TABLE 8:** Composition of the standard media (MS, Murashige and Skoog; B5, Gamborg; St, Standardi; Ha, -Harada) and the predicted optimized *R* medium together with output values obtained at validation experiment (mean ± standard deviation).

	**R**	**MS**	**B5**	**St**	**Ha**

**Macronutrients (mg L^–1^)**					

KNO_3_	**1604.18**	1900	2500	1800	1900
NH_4_NO_3_	**1297.09**	1650	–	400	1650
(NH_4_)_2_SO_4_	–	–	134	–	–
Ca(NO_3_)_2_ 4H_2_O	–	–	–	1200	–
CaCl_2_ 2H_2_O	**545.61**	440	150	–	440
MgSO_4_ 7H_2_O	**926.54**	370	250	360	370
KH_2_PO_4_	**375.63**	170	–	270	170
NaH2PO_4_ H2O	–	–	150	–	–

**Micronutrients (mg L^–1^)**					

MnSO_4_ 4H_2_O	**5.63**	22.30	13.20	1.0	25.00
ZnSO_4_ 7H_2_O	**2.97**	8.60	2.00	8.60	10.00
H_3_BO_3_	**5.07**	6.20	3.00	6.20	10.00
KI	**1.43**	0.83	0.75	0.08	–
CuSO_4_ 5H_2_O	**0.05**	0.025	0.025	0.025	0.025
Na_2_MoO_4_ 2H_2_O	**0.28**	0.25	0.25	0.25	0.25
CoCl_2_ 6H_2_O	**0.04**	0.025	0.025	0.025	–
FeSO_4_ 7H_2_O	**49.54**	27.85	27.85	27.85	27.85
Na_2_ EDTA 2H_2_O	**53.88**	37.25	37.25	37.25	37.25

**Vitamins (mg L^–1^)**					

Myo-inositol	100	100	100	100	500
Thiamine-HCl	0.1	0.1	10.0	4.0	0.5
Nicotinic-acid	0.5	0.5	1.0	–	5.0
Pyridoxine-HCl	0.5	0.5	1.0	–	0.5
Biotine	–	–	–	–	0.05
Folic acid	–	–	–	–	0.5
Glycine	2.0	2.0	–	–	2.0

**Outputs values for the validation experiment**					
**SN**	**3.6 ± 1.12**	3.60 ± 1.01	3.51 ± 1.10	4.10 ± 1.16	4.04 ± 0.82
**SL (cm)**	**2.69 ± 0.55^*b*^**	1.72 ± 0.40^*d*^	1.88 ± 0.35^*d*^	3.41 ± 1.20^*a*^	2.10 ± 0.60^*cd*^
**LA (cm^2^)**	**34.06 ± 10.29^*bc*^**	29.58 ± 7.62^*cd*^	21.43 ± 6.86^*e*^	43.05 ± 8.57^*a*^	38.39 ± 11.04^*ab*^
**SQ**	**4.51 ± 0.51^*a*^**	4.13 ± 0.7^*b*^	3.22 ± 0.60^*c*^	4.02 ± 0.27^ ab^	3.87 ± 0.46^*b*^
**BC**	**4.00 ± 0.00^*a*^**	4.00 ± 0.00^*a*^	3.00 ± 0.00^*c*^	3.60 ± 0.50^*b*^	4.00 ± 0.00^*a*^
**H**	**2.80 ± 0.46**	2.64 ± 0.68	2.80 ± 0.55	2.67 ± 0.53	2.60 ± 0.69

*Values with the same or without a letter are not significantly different at α = 0.001 (Tukey’s HSD Test). Predicted optimized R media composition and validated results are bolded. SN, shoot number; SL, shoot length; LA, leaf area; SQ, shoot quality; BC, basal callus; and H, hyperhydricity.*

Compared to the MS medium, the optimized *R* medium significantly improved (α < 0.001) the two responses selected as important in the optimization process (SQ and SL) with a weight of 10 and 9, respectively (100% desirability), but not the other outputs (Table 8).

Regarding the other media used, the optimized *R* medium was surpassed by the St medium in promoting the growth parameters SL and LA (α < 0.001; Table 8), however, *R* medium obtained a better value for the SQ (α < 0.001) with less formation of basal calluses and hyperhydricity. On the contrary, medium B5 promoted the lowest values of SQ, BC, SL, and LA (α < 0.001). Finally, the Ha medium promoted lower SQ and SL than *R* medium (α < 0.001; Table 8).

## Discussion

### ML as New Strategy to Predict Optimal Mineral Nutrition

Recently, algorithm-based approaches were introduced in plant tissue culture studies. Decision Trees, Chi-square Automatic Interaction Detector (CHAID), and adaptive regression splines were preferred to other ML methods such as ANNs, considering that ANNs generate “black box” models that are difficult to interpret and use (Olden et al., 2008; Akin et al., 2016, 2020). However, ANNs are powerful ML tools for plant tissue researchers, particularly when they are combined with other techniques that help in the interpretation of results or the use of the models (Gago et al., 2010b; Gallego et al., 2011). Some of their strengths are: (i) ANNs do not require a specific experimental design, so they can deal with incomplete factorial designs, trial-error series, or even historical data; (ii) they do not entail orthogonality or uniformity in the data; and (iii) subtle non-linear relationships in the data can be elucidated. The two major weakness of ANNs are the possibility of “overtraining” and the generation of “black box” models. The first limits the predictability and the second limits the possibility of using the model out of the computer used to generate it. Those limitations can be overcome by validating the model with unseen data before using it and combining the ANN model with other technology that allows knowledge to be extracted. Thus, systems that combine ANN with fuzzy logic or “neurofuzzy logic” systems allow for the obtaining of “gray box” models, providing sets of linguistic rules that help to generate knowledge about the process studied; and the combination of ANNs with GA is able to answer “How to get” questions to find the compromised solution to obtain simultaneously a set of desirable outputs. In the present work we take advantage of the use of two ML techniques, neurofuzzy logic and ANN-GA, to study the mineral nutrition of kiwiberry and address the development of an efficient *in vitro* protocol for this cultivar, as it has been carried out for other species by further innovative research groups (Zielińska and Kêpczyńska, 2013; Nezami-Alanagh et al., 2019).

The MS medium was the first and only medium described in the literature for kiwiberry *in vitro* culture (Seelye and Butcher, 1991; Matkowski and Przywara, 1995; Han et al., 2010). However, several authors have reported side effects and physiological disorders, such as shoot tip necrosis and hyperhydricity, when using it (Nowak et al., 2007; Bhojwani and Dantu, 2013; Nezami-Alanagh et al., 2018 and references therein). MS medium has also been considered supra optimal for other species of kiwifruit (Moncaleán et al., 1999, 2003), being necessary to reduce its composition by half or even more to improve performance (Monette, 1986; Akbas et al., 2007; Nasib et al., 2008).

Based on the salt composition of the MS medium, a reduced experimental design has been established (Tables 1, 2). MS medium includes a combination of 14 salts. It is almost impossible to develop a complete factorial design for all of them because of the number of treatments to be assayed (e.g., 14 factors at three levels give 4,782,969 treatments). Instead, MS media salts were grouped in to five factors, which were used to define a five-dimensional design space, including just 33 treatments.

The factors selection followed the strategy used by other authors to study the shoot quality of the hazelnut, raspberry, or apricot (Wada et al., 2013; Hand et al., 2014; Poothong and Reed, 2014; Kovalchuk et al., 2017). Factors were: (i) NH_4_NO_3_, (ii) KNO_3_, (iii) mesos, (iv) micros, and (v) iron (Reed et al., 2013a,b; Akin et al., 2017). The levels were chosen according to the maxima and minima found in the literature, in-house experience (Nezami-Alanagh et al., 2018, 2019), and the biological actions of the different nutrients (Hameg, 2019). This approach leads to more manageable and feasible research, ensuring adequate sampling of the design space (Niedz and Evens, 2006; Niedz and Evens, 2007).

The use of salts as factors creates the ion confounding effect, as has been demonstrated by some authors (Niedz and Evens, 2006), making it difficult to detect and explain the effects of individual ions on growth responses. However, it has been postulated that the specific control of some ions, such as K^+^, NH_4_^+^, and NO_3_^–^, is a critical aspect in the optimization of culture media (Akin et al., 2017). In order to study the effects of the specific ions on growth parameters and/or physiological disorders of *in vitro* kiwiberry culture, the ionic composition for the different treatments was calculated (Table 3) and modeled by a neurofuzzy logic software. For all parameters studied, the models showed high predictability (Train Set *R*^2^ > 70%) and good accuracy. Reading the simple “IF-THEN” rules allows the acquisition of knowledge about the ions that are critical for the response parameters of kiwiberry explants. Subsequently, the ANNs-GA modeling was also carried out using the salt database, which allowed for predicting of the mineral nutrients of an optimal *R* medium, specifically adapted to kiwiberry tissue culture.

### ML to Understand Shoot Growth Responses

Seven ions explain the variations in shoot number: NH_4_^+^, NO_3_^–^, SO_4_^2–^, BO_3_^–^, MoO_2_^2–^, Na^+^, and I^–^. Neurofuzzy rules (Table 6) state that a low MoO_2_^2–^ supplement (<0.0008 mM, Table 9) is necessary with all Na^+^ concentrations to promote a high number of shoots. Molybdate is generally added to the culture media up to 1 mM, and participates in NO_3_^–^ catabolism (George et al., 2008). The *R* medium designed here is also proposed as 0.001 mM.

**TABLE 9:** Ranges (mM) and meaning of the levels (low, mid, and high) after fuzzification process by neurofuzzy logic software according to the rules Tables 6, 7 for each response parameter.

**Inputs**	**Level**	**SN**	**SL**	**LA**	**SQ**	**BC**	**H**	**R**	**MS**	**St**	**Ha**	**B5**
NH_4_^+^	Low	4.12 < × < 12.37				4.12 < × < 8.25						
	Mid		–	–	–	8.25 < × < 16.49	–	16.20	20.61	5.00	20.61	1.01
	High	12.37 < × < 20.61				16.49 < × < 20.61						
NO_3_^–^	Low	6.00 < × < 22.71		6.00 < × < 22.71	6.00 < × < 14.35							
	Mid				14.35 < × < 31.06	–	–	32.07	39.41	32.96	39.41	24.73
	High	22.71 < × < 39.41		22.71 < × < 39.41	31.06 < × < 39.41							
K^+^	Low		2.19 < × < 12.37		2.19 < × < 12.37							
	Mid	–	–	–		–	–	18.64	20.05	19.79	20.05	24.73
	High		12.37 < × < 22.55	–	12.37 < × < 22.55	–	–					
Ca^2+^	–	–	–	–	–	–	–	3.71	2.99	5.08	2.99	1.02
Mg^2^+	Low		0.38 < × < 1.41									
	Mid	–	1.41 < × < 3.47	–	–	–	–	3.76	1.50	1.46	1.50	1.01
	High		3.47 < × < 4.50									
PO_4_^3–^	Low		0.31 < × < 1.17			0.31 < × < 1.17						
	Mid	–	1.17 < × < 2.89	–	–	1.17 < × < 2.89	–	2.76	1.25	1.98	1.25	1.09
	High		2.89 < × < 3.75			2.89 < × < 3.75						
SO_4_^2–^	Low	0.49 < × < 2.85			0.49 < × < 1.67	0.49 < × < 1.67	0.49 < × < 2.85					
	Mid		–	–	2.85 < × < 4.02	2.85 < × < 4.02		3.97	1.73	1.60	1.73	2.21
	High	2.85 < × < 5.20			4.02 < × < 5.20	4.02 < × < 5.20	2.85 < × < 5.20					
Cl^–^	Low			1.50 < × < 9.73			1.50 < × < 9.73					
	Mid	–	–		–	–		7.42	5.99	0.00	5.99	2.04
	High			9.73 < × < 17.96			9.73 < × < 17.96					
Fe^2+^	Low		0.10 < × < 0.30		0.10 < × < 0.30							
	Mid	–		–		–	–	0.18	0.10	0.10	0.10	0.10
	High		0.30 < × < 0.50		0.30 < × < 0.50							
BO^3–^	Low	0.01 < × < 0.08	0.01 < × < 0.05		0.01 < × < 0.05		0.01 < × < 0.08					
	Mid		0.05 < × < 0.12	–	0.05 < × < 0.12	–		0.08	0.10	0.10	0.16	0.05
	High	0.08 < × < 0.15	0.12 < × < 0.15		0.12 < × < 0.15		0.08 < × < 0.15					
Mn^2+^		–	–	–	–	–	–	0.03	0.13	0.01	0.15	0.08
Zn^2+^		–	–	–	–	–	–	0.01	0.03	0.03	0.03	0.01
Cu^2+^		–	–	–	–	–	–	0.0002	0.0001	0.0001	0.0001	0.0001
MoO_2_^2–^	Low	0.0001 < × < 0.0008										
	Mid		–	–	–	–	–	0.001	0.001	0.001	0.001	0.001
	High	0.0008 < × < 0.0016										
Na^+^	Low	0.20 < × < 0.60		0.20 < × < 0.60								
	Mid		–		–	–	–	0.29	0.10	0.20	0.20	1.29
	High	0.60 < × < 1.00		0.60 < × < 1.00								
Co^2+^		–	–	–	–	–	–	0.0002	0.0001	0.0019	0.0	0.0001
I^–^	Low	0.001 < × < 0.002					0.001 < × < 0.004					
	Mid	0.002 < × < 0.06	–	–	–	–		0.008	0.005	0.0	0.0	0.005
	High	0.006 < × < 0.008					0.004 < × < 0.008					
EDTA^–^		–	–	–	–	–	–	0.14	0.1	0.1	0.1	0.1
Na^+^	Low	0.20 < × < 0.60		0.20 < × < 0.60								
	Mid		–		–	–	–	0.29	0.10	0.20	0.20	1.29
	High	0.60 < × < 1.00		0.60 < × < 1.00								
Co^2+^		–	–	–	–	–	–	0.0002	0.0001	0.0019	0.0	0.0001
I^–^	Low	0.001 < × < 0.002					0.001 < × < 0.004					
	Mid	0.002 < × < 0.06	–	–	–	–		0.008	0.005	0.0	0.0	0.005
	High	0.006 < × < 0.008					0.004 < × < 0.008					
EDTA^–^		–	–	–	–	–	–	0.14	0.1	0.1	0.1	0.1

*No ranges (–) are included in those ions not modeled. Also, the optimized R designed by ANNs-GA and MS (Murashige and Skoog), St (Standardi), Ha (Harada), and B5 (Gamborg) medium ion composition (mM) are included for easy comparison. SN, shoot number; SL, shoot length; LA, leaf area; SQ, shoot quality; BC, basal callus; and H, hyperhydricity.*

Na^+^ is considered a functional but inessential element, except for C4 plants, due to its relationship with CO_2_ fixation (George et al., 2008). Sotiropoulos and Dimassi (2004) have reported the beneficial effect of NaCl in the range of 10–20 mM on the *in vitro* proliferation of *Actinidia deliciosa*. Sodium is part of different salts added to the culture media, so it is usually difficult to establish its specific needs since it is affected by the ion confounding effect. However, the neurofuzzy logic model established that the Na^+^ concentration within the limits of the study (0.2–1 mM) is adequate to obtain a high number of shoots for kiwiberry, especially when the molybdate is in low concentration (<0.0008 mM, Table 9).

Iodine has not been recognized as an essential mineral for plant nutrition, but it is included in almost 65% of the media (George et al., 2008). Chée (1986) has suggested that I^–^ interferes with lateral auxin transport and/or facilitates its catabolism. Generally, iodine is added to the culture media through the KI salt. The effect of adding KI to the media is controversial in the literature. Some authors (Da Silva et al., 2017) have pointed out that KI accelerates the development of plants. However, KI is not added to various media specifically designed for woody plants, such as DKW (Driver and Kuniyuki, 1984) or WPM (Lloyd and McCown, 1980). It is also not incorporated into the Ha medium, which is designed for kiwi. Neurofuzzy logic suggests that medium-high concentrations of iodine (>0.006 mM), whatever the concentration of SO_4_^2–^, contributes to improving the number of shoots.

Sulfur is an essential component and plays an important role as ligand through the -SH groups. Most culture media contain this ion in the range of 1–2.5 mM. The available form of sulfur in plants is as SO_4_^2–^ ion and it is supplied in plant tissue cultures combined with other essential elements (Mg, Mn, Zn, Cu, and Fe) in at least five different salts, therefore its own role in mineral nutrition is still poorly understood due to the ion confounding effect. The neurofuzzy ML tool allows us to deduce its key role not only in the number of shoots, but in all the quality parameters studied (see below). Interestingly, the two most frequently cited media for woody plants, WPM and DKW, contain a high concentration of sulfate (7.45 and 12.39 mM, respectively). Our results demonstrate that the SO_4_^2–^, *per se*, in concentrations higher than 2.85 mM (Table 9), is essential to improve the number of shoots in woody fruit tree plants, which is in agreement with other authors (Poothong and Reed, 2014) who have shown that the high level of mesos and micros, including MgSO_4_, MnSO_4_, ZnSO_4_, and CuSO_4_, promotes this effect on red raspberries.

Finally, the neurofuzzy model established the positive effect of the interaction among NH_4_^+^ × NO_3_^–^ × BO_3_^–^ on the regeneration of new shoots (SN). BO_3_^–^ is generally supplemented in a range of 0.05–0.1 mM, being toxic at higher concentrations (0.185 mM; Bowen et al., 1979). As I^–^, BO_3_^–^ also stimulates auxin catabolism and increases its translocation (George et al., 2008), the excess of boron counteracts these important morphogenetic PGRs. Other studies showed that boron deficiency affects cell elongation more than cell division in plant growth (Martín-Rejano et al., 2011). High concentrations of BO_3_^–^, within the range of the study (>0.08 mM; to avoid possible toxicity), should be added to the media for obtaining high SN, independently of the levels of both nitrogen and ammonium ions.

Nitrogen sources, mainly in form of NH_4_^+^ and NO_3_^–^, are constituents of proteins, nucleic acid, and chlorophyll, being essential to plant life (George et al., 2008). In general, in media design, NO_3_^–^ and NH_4_^+^ are combined as the latter acidifies the medium. The addition of NO_3_^–^ counteracts this effect buffering the pH (George et al., 2008). Although different NO_3_^–^/NH_4_^+^ ratios have been tested, most media, as MS, have twice as much nitrate as ammonium, as a useful control of media pH. Furthermore, while high NO_3_^–^ levels are non-toxic, high NH_4_^+^ levels promote physiological abnormalities such as hyperhydricity (Lips et al., 1990; Nezami-Alanagh et al., 2019). The neurofuzzy logic model points out the importance of both nitrogen ions, in combination with BO_3_^–^ whatever its concentration, suggesting that the main role of NH_4_^+^ and NO_3_^–^ could also be related with their function as pH media control, rather than only nutrients.

The shoot elongation of *A. arguta* was significantly affected by five ions (Table 9). With the exception of BO_3_^–^, the ions that explain the variability in the elongation of the shoots are different from those that intervene in the appearance of new shoots. The model pinpointed the key positive effect of Mid Mg^2+^ (1.41 < *X* < 3.47 mM), Low Fe^2+^ (<0.3 mM), High K^+^ (>12.37 mM), and Mid BO_3_^–^ (0.05 < *X* < 0.12 mM) (Table 9) on shoot length growth. Finally, Mid PO_4_^3–^ (1.17 < *X* < 2.89) reduced the SL.

Several studies have shown the importance of mesos salts on shoot length. Thus, it has been described that for apricot this parameter is affected by K_2_SO_4_ levels (Kovalchuk et al., 2017). Moreover, High mesos (MgSO_4_⋅7H_2_O, and KH_2_PO_4_) and Low iron are required to enhance red raspberries’ shoot length (Poothong and Reed, 2014, 2015). Accordingly, our results (Table 8), support the same pattern for another fruit tree species, *A. arguta*. The cause-effect of the specific ions is thus demonstrated, avoiding the ion confounding effect.

Within the limits of the study, to obtain a large leaf area, a combination of High NO_3_^–^ (>22.71 mM) and Low Na^+^ (<0.60 mM) concentrations (Table 6, rule 33) should be added to the media. Some authors have demonstrated that High KNO_3_ and NH_4_NO_3_ levels improve the number and size of leaves in pear genotypes (Ibrahim et al., 2008; Reed et al., 2013a). Also, leaf area can be affected by NO_3_^–^ factor as has been shown for red raspberry (Poothong and Reed, 2016). All these findings agree with our results (Table 8).

### ML to Understand Morphophysiological Disorders and Shoot Quality Responses

Morphophysiological disorders in *in vitro* cultures are caused by a wide variety of factors (Hazarika, 2006) and avoiding them is one of the greatest challenges in shoots micropropagation. In this study, important abnormalities such as BC and hyperhydricity were observed (Figure 3) and neurofuzzy logic was able to determine the ions that are directly related to the appearance of them.

The effect of three ions on the development of basal callus were pointed out; PO_4_^3–^ × NH_4_^+^ and SO_4_^2–^ were included in the factors nitrogen (NH_4_NO_3_) and mesos (MgSO_4_). The model highlighted the positive effect of Mid-High PO_4_^3–^ (>1.17 mM) to favor the production of healthy shoots with moderate or absent callus formation (Figure 2B), particularly when Low NH_4_^+^ (<8.25 mM) are used, in agreement with other reports (Niedz and Evens, 2007; Kovalchuk et al., 2018). If Low PO_4_^3–^ is added to the media, necrosis and the formation of big basal callus are significantly promoted, whatever the NH_4_^+^ concentration. Finally, SO_4_^2–^ at any concentration tested in this space of design reduces the basal callus formation, generating healthy shoots (High BC). The beneficial effect of high concentrations of these ions on callus formation has been previously demonstrated for other species, such as pear (Reed et al., 2013b), raspberries (Poothong and Reed, 2014), or hazelnut (Akin et al., 2017, 2020). MS medium promotes higher callus formation than WPM and DKV in pistachio culture (Nezami-Alanagh et al., 2019), probably due to its lower and higher levels of NH_4_^+^ and SO_4_^2–^, respectively.

Hyperhydricity is one of the main morpho-physiological disorders in the micropropagation of plants that has been associated with alterations in mineral composition, hormonal imbalances, or the use of gelling agents (Pâques, 1991; Hazarika, 2006). Within the range of the study, kiwiberry also showed hyperhydricity for some treatments (Figure 3B). The hyperhydricity was caused by the interaction of four ions: Cl^–^ × I^–^ and SO_4_^2–^ × BO_3_^–^. The combination of High I^–^ (>0.004 mM) and Low BO_3_^–^ (<0.08 mM) concentrations in the medium avoided hyperhydricity formation, whatever the concentration of Cl^–^ and SO_4_^2–^, respectively. High percentages of hyperhydricity in *Prunus* and *Dianthus caryophyllus* cultures have been associated with high concentrations of Cl^–^ in the culture medium (Quoirin and Lepoivre, 1977; Dantas et al., 2001). In contrast, high concentrations of mesos (CaCl_2_, KH_2_PO_4_, and MgSO_4_) in pear cultures reduce hyperhydricity (Reed et al., 2013b).

The shoot quality parameter integrates, in some way, both the growth parameters, and the absence of abnormalities and physiological disorders. The neurofuzzy logic model selected five ions as critical factors for the quality of the shoots: K^+^, SO_4_^2–^, Fe^2+^, BO_3_^–^, and NO_3_^–^. All of them contribute in a way to the growth parameters (SN, SL, or LA), but some are also involved in the appearance of abnormalities as SO_4_^2–^ (BC and H) or BO_3_^–^ (H).

Rules reveal that good shoot quality is achieved when a High K^+^ (>12.35 mM) concentration is supplied to the medium along with concentrations of Fe^2+^ and NO_3_^–^ within the ranges of 0.10–0.30 mM and 14.35–31.06 mM, respectively, in agreement with other authors for other cultivars (Akin et al., 2017; Kovalchuk et al., 2017) and also with the predictions for the growth parameters of kiwiberry. Medium SO_4_^2–^ (2.85–4.02 mM) concentration is also necessary, since it promotes a high number of shoots and plantlets with low callus formation. Finally, BO_3_^–^ must be in 0.05–0.12 mM due to its effect on the number of shoots and hyperhydricity (Table 9).

### ML to Predict Optimal Salt Composition and Experimental Validation

Different ML models based on artificial neural networks, fuzzy logic, genetic algorithms, or gene expression programming algorithms has been previously employed with success for predicting optimal *in vitro* culture media of fruit tree species such as *Prunus* (Nezami-Alanagh et al., 2014; Arab et al., 2016, 2018), pear (Jamshidi et al., 2016, 2019), or pistachio rootstocks (Nezami-Alanagh et al., 2017). In this study, ML tools, including ANNs-GA were selected to build a model based on salt composition of culture media for each growth parameter. All of them have good predictability (Train and Test Set *R* > 70%). The utility of GA allows the estimation of the best combination of salts to obtain a set of desired values for each parameter (maximal growth parameters). The model predicts for a medium of optimal composition (*R* medium) values of SN, SL, LA, and SQ of 4.8 shoots, 2.6 cm, 39.5 cm^2^, and 4.4, respectively.

The experimental values obtained for kiwiberry culture using the *R* medium composition validate those predicted by the model. Even more, the comparison of the results obtained with the *R* medium and the four media (MS, St, Ha, and B5), used as controls in the validation experiment, shows that the optimized *R* medium outperforms the others in terms of SL, LA, and SQ. However, the SN parameter appears to be a bit overestimated (4.8 versus 3.6). Only, St basal medium promoted statistically significant (α < 0.001) larger SL and LA.

Differences in mineral composition among all media (Table 9) reveals that St is the poorest media in NH_4_^+^ and Cl^–^ but rich in Ca^2+^, as is other media used for woody plants such as DKW (Driver and Kuniyuki, 1984) and WPM (Lloyd and McCown, 1980), while B5 included low nitrogen but also the lowest mesos (Ca^2+^, Mg^2+^, and PO_4_^3–^) concentration among the media tested, but the highest Na^+^ (Table 9; Hameg et al., 2018). However, the optimized *R* medium adjusted at $\frac{3}{4}$ total nitrogen of MS content, but increased 2× all mesos (Ca^2+^, Mg^2+^, PO_4_^3–^, and SO_4_^2–^) and also increased almost all micros (Cl^–^, Cu^2+^, Na^+^, Co^2+^, and I^–^) and iron (Fe^2+^ and EDTA^–^) with respect to MS (Table 9). The increasing levels of micronutrients over the level in MS promoted cell growth and morphogenesis in some species (George et al., 2008). With those adjustments, the medium *R* promoted better results than MS, particularly shoot quality. In conclusion, it is clear that it is the interactions among the ions, rather than their independent effect, that caused the described results. Thus, it is multivariable analysis, rather than single-factor analysis, that is required to really understand media component relationships. Finally, another important fact is that those media (St, Ha, and B5) included different vitamin contents and glycine, not included in this optimization. Furthermore, the PGR effect on organogenesis and growth was not studied, because all media were supplemented with the same PGRs.

## Conclusion

The suitability of computer-based tools, such as DOE and ML, as a new strategy to design tissue culture media for kiwiberry has been stated. DOE allowed the plant cell tissue researchers to perform well sampled and efficient experiments in order to save time and plant material. ML tools allowed for the extraction of information to clarify the complex non-linear interactions between variables and understand the effects of single ions on growth parameters and morpho-physiological disorders. A new medium, named *R* medium, was established with excellent results. The designed *R* medium differs from MS by reducing up to 20% nitrogen, increasing almost 200% mesos, 100% micros, and 50% iron factor concentrations and performs better for kiwiberry. The *R* medium also performs better than the B5, Ha, and St media, since although some of them have slight advantages in terms of growth parameters, they also promote more physiological disorders. The *R* medium could be improved considering the effects of other key components of the media that have not been studied in this work, such as vitamins, PGR, or organic compounds, particularly glycine, that can modulate the effect of ions. They need further additional research.

## Data Availability Statement

The original contributions generated for this study are included in the article/Supplementary Material, further inquiries can be directed to the corresponding author.

## Author Contributions

RH and TA performed micropropagation experiments. RH, TA, and MB performed the statistical analysis. ML and PG contributed to machine learning models supervision. MB and PG conceived and designed the experiments. All authors equally contributed to the discussions for data interpretation and drawing the conclusions. All authors contributed to the writing and agreed to the published version of the manuscript.

## Conflict of Interest

The authors declare that the research was conducted in the absence of any commercial or financial relationships that could be construed as a potential conflict of interest.

## Supplementary Material

Supplementary Figure 1 Determination coefficient (*R*^2^) between the predicted values from the neurofuzzy logic versus experimental values for each factor expressed as determination coefficient (*R*^2^): **(A)** SN, shoot number; **(B)** SL, shoot length; **(C)** LA, leaf area; **(D)** SQ, shoot quality; **(E)** BC, basal callus formation; and **(F)** H, hyperhydricity.

Supplementary Figure 2 Examples of graphical representation of the fuzzyfication process developed by neurofuzzy logic per NO_3_^–^ **(A)** and K^++^ **(B)** and their respective domains Low–Mid–High and Low–High.

Supplementary Table 1 Mineral nutrients’ (expressed as salt concentrations) composition of the different culture media based on the five-factor experimental design (0–33) and response values of the parameters (mean and standard deviation) used to characterize plant growth. Original medium composition (bold) used as control. SN, shoot number; SL, shoot length; LA, leaf area; SQ, shoot quality; BC, basal callus; and H, hyperhydricity.

Supplementary Table 2 Artificial neural network model train set *R*^2^ and test set *R*^2^. MSE (Mean Squared Error).
