# Supplementary material for: Modeling and Optimizing Culture Medium Mineral Composition for in vitro Propagation of Actinidia arguta
Source: Front Plant Sci. 2020 Dec 23;11:554905. doi: 10.3389/fpls.2020.554905 (PMC7785940; doi:10.3389/fpls.2020.554905)
Supplement: Supplementary Figure 1 — Determination coefficient (R2) between the predicted values from the neurofuzzy logic versus experimental values for each factor expressed as determination coefficient (R2): (A) SN, shoot number; (B) SL, shoot length; (C) LA, leaf area; (D) SQ, shoot quality; (E) BC, basal callus formation; and (F) H, hyperhydricity. [file Table_1.DOCX]

SUPLEMMENTARY TABLE 1. Macro and micronutrients (expressed as salt concentrations) composition of the different culture media based on the five-factor experimental design (0-33) and response values of the parameters (average and standard error) used to characterize plant growth. Original medium composition (bold) used as control.

|  | **Salts (mg L^-1^)** | | | | | | | | | | | | | | **Growth parameters** | | | **Quality parameters** | | |
| --- | --- | --- | --- | --- | --- | --- | --- | --- | --- | --- | --- | --- | --- | --- | --- | --- | --- | --- | --- | --- |
| **Media** | **KNO_3_** | **NH_4_NO_3_** | **CaCl_2_•2H_2_O** | **Mg SO_4_•7H_2_O** | **KH_2_PO_4_** | **MnSO_4_•4H_2_O** | **ZnSO_4_•7H_2_O** | **H_3_BO_3_** | **KI** | **CuSO_4_•5H_2_O** | **Na_2_MoO_4_•2H_2_O** | **CoCl_2_•6H_2_O** | **Na_2_•EDTA•2H_2_O** | **FeSO_4_•7H_2_O** | **SN** | **SL (cm)** | **LA (cm^2^)** | **SQ** | **BC** | **H** |
| **1** | 1250 | 838 | 727 | 611 | 280.9 | 33.45 | 12.90 | 9.30 | 1.25 | 0.038 | 0.375 | 0.0375 | 113.2 | 84.7 | 5.0±0.8 | 1.6±0.4 | 20.6±6.3 | 3.8±0.7 | 4.0±0.0 | 2.9±0.5 |
| **2** | 1259 | 1650 | 1139 | 957 | 439.9 | 2.23 | 0.86 | 0.62 | 0.08 | 0.003 | 0.025 | 0.0025 | 37.3 | 27.9 | 5.4±2.7 | 1.7±0.3 | 40.7±10.9 | 3.2±1.2 | 4.0±0.0 | 1.8±0.9 |
| **3** | 1900 | 1650 | 1042 | 876 | 402.5 | 33.45 | 12.90 | 9.30 | 1.25 | 0.038 | 0.375 | 0.0375 | 40.2 | 30.1 | 2.7±0.8 | 2.3±0.8 | 26.1±12.8 | 4.4±0.7 | 4.0±0.0 | 2.8±0.5 |
| **4** | 447 | 337 | 1320 | 1110 | 510.0 | 21.59 | 8.32 | 6.00 | 0.80 | 0.024 | 0.242 | 0.0242 | 68.5 | 51.2 | 5.8±2.6 | 1.9±0.6 | 12.0±4.0 | 3.5±0.5 | 4.0±0.0 | 1.8±1.0 |
| **5** | 190 | 1485 | 110 | 93 | 42.5 | 2.23 | 0.86 | 0.62 | 0.08 | 0.003 | 0.025 | 0.0025 | 59.6 | 44.6 | 5.8±1.6 | 1.2±0.3 | 7.1±3.4 | 3.1±0.5 | 4.0±0.0 | 3.0±0.0 |
| **6** | 1900 | 330 | 413 | 347 | 159.4 | 2.23 | 0.86 | 0.62 | 0.08 | 0.003 | 0.025 | 0.0025 | 186.3 | 139.3 | 2.6±1.1 | 1.2±0.4 | 6.8±3.2 | 2.5±0.9 | 4.0±0.0 | 3.0±0.0 |
| **7** | 1900 | 330 | 413 | 347 | 159.4 | 2.23 | 0.86 | 0.62 | 0.08 | 0.003 | 0.025 | 0.0025 | 186.3 | 139.3 | 2.2±1.2 | 1.4±0.5 | 7.8±4.4 | 2.2±0.9 | 4.0±0.0 | 3.0±0.0 |
| **8** | 1900 | 660 | 110 | 93 | 42.5 | 33.45 | 12.90 | 9.30 | 1.25 | 0.038 | 0.375 | 0.0375 | 37.3 | 27.9 | 7.9±2.7 | 1.1±0.2 | 8.7±3.9 | 2.8±0.5 | 3.5±0.5 | 2.9±0.3 |
| **9** | 190 | 1339 | 110 | 93 | 42.5 | 31.26 | 12.06 | 8.69 | 1.16 | 0.035 | 0.351 | 0.0351 | 186.3 | 139.3 | 4.1±1.5 | 0.7±0.2 | 3.1±1.7 | 1.7±0.5 | 4.0±0.0 | 3.0±0.0 |
| **10** | 1900 | 1650 | 1320 | 1110 | 510.0 | 8.63 | 3.33 | 2.40 | 0.32 | 0.010 | 0.097 | 0.0097 | 186.3 | 139.3 | 2.2±1.1 | 1.3±0.5 | 9.2±3.6 | 2.3±1.0 | 4.0±0.0 | 2.2±1.0 |
| **11** | 698 | 337 | 1320 | 1110 | 510.0 | 2.23 | 0.86 | 0.62 | 0.08 | 0.003 | 0.025 | 0.0025 | 186.3 | 139.3 | 3.7±1.1 | 1.0±0.3 | 6.0±2.1 | 1.7±0.7 | 4.0±0.0 | 1.9±1.0 |
| **12** | 190 | 1339 | 110 | 93 | 42.5 | 31.26 | 12.06 | 8.69 | 1.16 | 0.035 | 0.351 | 0.0351 | 186.3 | 139.3 | 4.2±1.5 | 0.8±0.2 | 3.8±1.2 | 1.5±0.5 | 3.1±0.2 | 3.0±0.0 |
| **13** | 190 | 1327 | 1314 | 1105 | 507.7 | 28.77 | 11.09 | 8.00 | 1.07 | 0.032 | 0.323 | 0.0323 | 37.3 | 27.9 | 4.8±1.0 | 1.7±0.4 | 17.0±5.5 | 3.6±0.7 | 4.0±0.0 | 1.9±0.7 |
| **14** | 190 | 653 | 110 | 93 | 42.5 | 2.23 | 0.86 | 0.62 | 0.08 | 0.003 | 0.025 | 0.0025 | 186.3 | 139.3 | 3.1±1.4 | 0.7±0.3 | 3.1±1.3 | 1.0±0.0 | 3.0±0.0 | 3.0±0.0 |
| **15** | 190 | 330 | 1175 | 988 | 453.9 | 33.45 | 12.90 | 9.30 | 1.25 | 0.038 | 0.375 | 0.0375 | 156.5 | 117.0 | 1.9±0.8 | 1.1±0.3 | 4.1±1.8 | 1.3±0.6 | 4.0±0.0 | 2.9±0.3 |
| **16** | 635 | 1650 | 110 | 93 | 42.5 | 33.45 | 12.90 | 9.30 | 1.25 | 0.038 | 0.375 | 0.0375 | 37.3 | 27.9 | 6.0±1.2 | 1.5±0.4 | 14.0±3.4 | 3.1±0.6 | 3.9±0.2 | 2.6±0.7 |
| **17** | 1223 | 1014 | 836 | 703 | 323.0 | 3.17 | 1.22 | 0.88 | 0.12 | 0.004 | 0.036 | 0.0036 | 131.8 | 98.5 | 1.9±1.1 | 1.2±0.4 | 8.7±2.3 | 2.3±0.9 | 4.0±0.0 | 3.0±0.0 |
| **18** | 1438 | 330 | 110 | 93 | 42.5 | 2.23 | 0.86 | 0.62 | 0.08 | 0.003 | 0.025 | 0.0025 | 37.3 | 27.9 | 6.1±1.4 | 1.3±0.3 | 8.8±2.8 | 2.9±0.5 | 2.0±0.0 | 2.9±0.3 |
| **19** | 1900 | 501 | 1320 | 1110 | 510.0 | 27.21 | 10.49 | 7.56 | 1.01 | 0.031 | 0.305 | 0.0305 | 186.3 | 139.3 | 2.9±1.5 | 1.7±0.7 | 14.5±6.4 | 3.4±1.0 | 4.0±0.0 | 2.7±0.8 |
| **20** | 190 | 1650 | 1048 | 881 | 404.8 | 2.23 | 0.86 | 0.62 | 0.08 | 0.003 | 0.025 | 0.0025 | 166.1 | 124.2 | 1.9±0.9 | 1.5±0.6 | 6.1±3.7 | 2.1±0.7 | 4.0±0.0 | 2.9±0.3 |
| **21** | 1900 | 1650 | 394 | 332 | 152.4 | 33.45 | 12.90 | 9.30 | 1.25 | 0.038 | 0.375 | 0.0375 | 178.1 | 133.1 | 1.1±0.3 | 1.7±0.3 | 5.7±3.4 | 2.3±1.0 | 4.0±0.0 | 3.0±0.0 |
| **22** | 1438 | 330 | 110 | 93 | 42.5 | 33.45 | 12.90 | 9.30 | 1.25 | 0.038 | 0.375 | 0.0375 | 186.3 | 139.3 | 4.3±0.9 | 1.0±0.2 | 4.1±1.4 | 1.7±0.5 | 1.0±0.0 | 3.0±0.0 |
| **23** | 1438 | 330 | 110 | 93 | 42.5 | 33.45 | 12.90 | 9.30 | 1.25 | 0.038 | 0.375 | 0.0375 | 186.3 | 139.3 | 3.2±1.3 | 1.0±0.3 | 4.0±1.6 | 1.7±0.5 | 1.2±0.7 | 3.0±0.0 |
| **24** | 1900 | 1650 | 110 | 93 | 42.5 | 2.23 | 0.86 | 0.62 | 0.08 | 0.003 | 0.025 | 0.0025 | 122.9 | 91.9 | 3.1±1.5 | 1.3±0.4 | 4.7±2.0 | 1.9±0.8 | 4.0±0.0 | 2.6±0.9 |
| **25** | 660 | 1650 | 667 | 561 | 257.6 | 20.96 | 8.08 | 5.83 | 0.78 | 0.024 | 0.235 | 0.0235 | 115.6 | 86.4 | 3.5±0.9 | 2.2±0.9 | 16.2±5.3 | 3.7±0.7 | 4.0±0.0 | 2.9±0.5 |
| **26** | 1447 | 330 | 1320 | 1110 | 510.0 | 33.45 | 12.90 | 9.30 | 1.25 | 0.038 | 0.375 | 0.0375 | 37.3 | 27.9 | 2.3±1.0 | 1.9±0.8 | 21.7±10.3 | 4.2±0.8 | 3.6±0.5 | 2.8±0.4 |
| **27** | 1900 | 396 | 1320 | 1110 | 510.0 | 2.30 | 0.89 | 0.64 | 0.09 | 0.003 | 0.026 | 0.0026 | 74.5 | 55.7 | 4.1±1.8 | 2.0±0.6 | 28.5±8.5 | 3.9±0.9 | 4.0±0.0 | 1.4±0.7 |
| **28** | 1900 | 396 | 1320 | 1110 | 510.0 | 2.30 | 0.89 | 0.64 | 0.09 | 0.003 | 0.026 | 0.0026 | 74.5 | 55.7 | 4.3±1.5 | 1.9±0.6 | 29.9±13.8 | 4.1±0.8 | 4.0±0.0 | 1.8±0.7 |
| **29** | 1447 | 330 | 1320 | 1110 | 510.0 | 33.45 | 12.90 | 9.30 | 1.25 | 0.038 | 0.375 | 0.0375 | 37.3 | 27.9 | 2.7±1.0 | 1.8±1.1 | 18.9±9.3 | 3.5±1.2 | 3.7±0.5 | 2.4±0.8 |
| **30** | 190 | 521 | 1030 | 866 | 397.8 | 2.23 | 0.86 | 0.62 | 0.08 | 0.003 | 0.025 | 0.0025 | 37.3 | 27.9 | 6.6±2.4 | 1.1±0.3 | 9.3±3.9 | 1.4±0.5 | 4.0±0.0 | 1.0±0.0 |
| **31** | 1370 | 1194 | 394 | 332 | 152.4 | 15.34 | 5.92 | 4.27 | 0.57 | 0.017 | 0.172 | 0.0172 | 37.3 | 27.9 | 3.9±1.4 | 2.6±0.9 | 28.3±9.3 | 4.7±0.5 | 4.0±0.0 | 2.7±0.5 |
| **32** | 190 | 330 | 352 | 296 | 136.0 | 30.33 | 11.70 | 8.43 | 1.13 | 0.034 | 0.340 | 0.0340 | 37.3 | 27.9 | 4.1±1.7 | 1.2±0.3 | 5.7±2.9 | 2.4±0.8 | 4.0±0.0 | 2.9±0.3 |
| **33** | 643 | 1650 | 1320 | 1110 | 510.0 | 33.45 | 12.90 | 9.30 | 1.25 | 0.038 | 0.375 | 0.0375 | 186.3 | 139.3 | 2.6±2.1 | 1.5±0.5 | 10.4±5.5 | 2.6±0.8 | 4.0±0.0 | 2.4±0.9 |
| **MS** | **1900** | **1650** | **440** | **370** | **170** | **22,30** | **8,60** | **6,20** | **0,83** | **0,025** | **0,250** | **0,0250** | **37,3** | **27.9** | **3.9±1.3** | **1.7±0.4** | **28.7±9.3** | **4.1±0.4** | **4.0±0.0** | **2.6±0.6** |
