# Supplementary material for: Modeling and Optimizing Culture Medium Mineral Composition for in vitro Propagation of Actinidia arguta
Source: Front Plant Sci. 2020 Dec 23;11:554905. doi: 10.3389/fpls.2020.554905 (PMC7785940; doi:10.3389/fpls.2020.554905)
Supplement: Supplementary Figure 2 — Examples of graphical representation of the fuzzyfication process developed by neurofuzzy logic per NO3– (A) and K++ (B) and their respective domains Low–Mid–High and Low–High. [file Table_2.DOCX]

**SUPPLEMENTARY TABLE 2.** Artificial neural network model train set R^2^ and test set R^2^.

| **Outputs** | **Train set R^2^** | **Test set R^2^** |
| --- | --- | --- |
| **Shoot quality** | 96.59 | 79.23 |
| **Shoot number** | 82.56 | 76.99 |
| **Shoot length** | 79.50 | 78.42 |
| **Leaf area** | 92.21 | 91.64 |
