# Supplementary figures and images for: Modeling and Optimizing Culture Medium Mineral Composition for in vitro Propagation of Actinidia arguta
Source: Front Plant Sci. 2020 Dec 23;11:554905. doi: 10.3389/fpls.2020.554905 (PMC7785940; doi:10.3389/fpls.2020.554905)

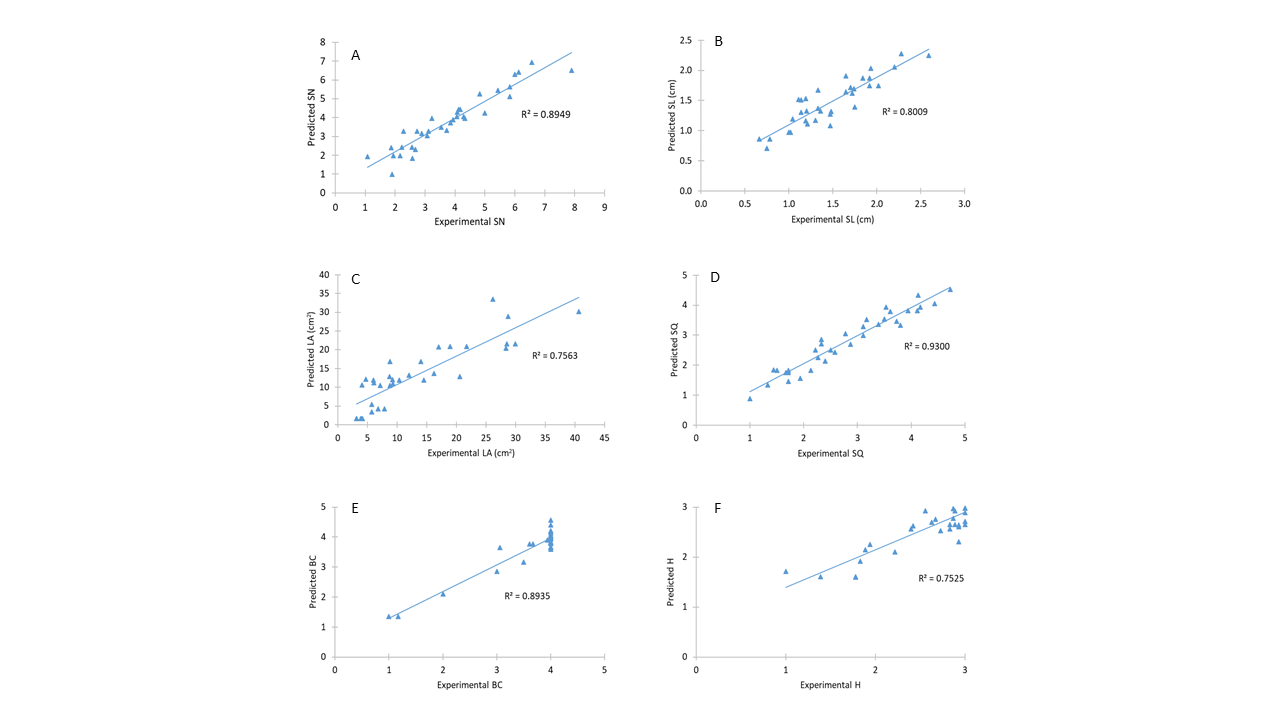

Supplement: Supplementary Table 1 — Mineral nutrients’ (expressed as salt concentrations) composition of the different culture media based on the five-factor experimental design (0–33) and response values of the parameters (mean and standard deviation) used to characterize plant growth. Original medium composition (bold) used as control. SN, shoot number; SL, shoot length; LA, leaf area; SQ, shoot quality; BC, basal callus; and H, hyperhydricity. [file Image_1.TIF]

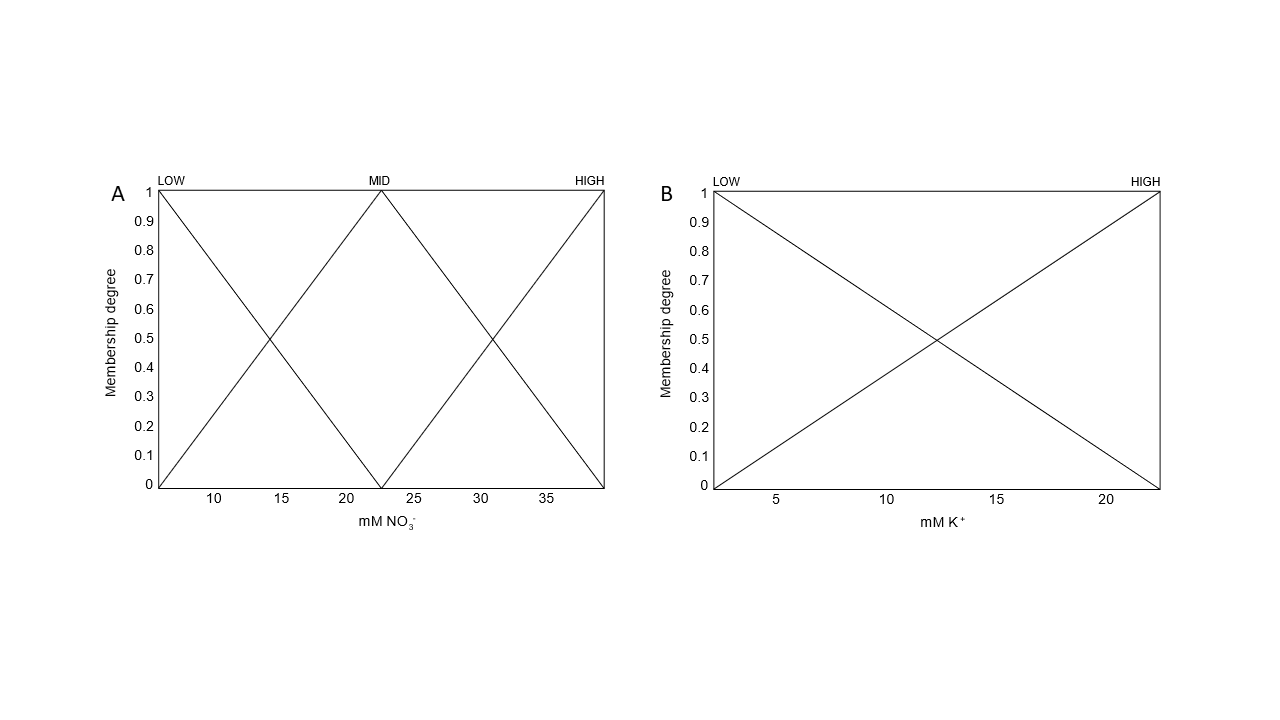

Supplement: Supplementary Table 2 — Artificial neural network model train set R2 and test set R2. MSE (Mean Squared Error). [file Image_2.TIF]
